# Cationized gelatin nanosphere-mediated release-controlled RNA interference targeting mTORC1 protects against intervertebral disc degeneration

**DOI:** 10.1016/j.bioactmat.2026.09.033

**Published:** 2026-10-11

**Authors:** Yoshiaki Hiranaka, Takashi Yurube, Naotoshi Kumagai, Yoshiki Takeoka, Yutaro Kanda, Kohei Kuroshima, Masahiko Furuya, Daisuke Nakagawa, Takahiro Kitano, Akihiro Miyajima, Taichi Washisaka, Kenichiro Kakutani, Ryosuke Kuroda, Yasuhiko Tabata

**Affiliations:** a Department of Orthopaedic Surgery, Kobe University Graduate School of Medicine, Kobe, Japan; b Department of Regeneration Science and Engineering, Institute for Life and Medical Sciences, Kyoto University, Kyoto, Japan; c Department of Orthopedic Surgery, Iwate Medical University School of Medicine, Yahaba, Japan; d Department of Plastic and Reconstructive Surgery, Graduate School of Medicine, Kyoto University, Kyoto, Japan; e Department of Chemical Engineering, Faculty of Engineering, Chulalongkorn University, Bangkok, Thailand

**Keywords:** Intervertebral disc degeneration, Cationized gelatin nanospheres, Controlled release, siRNA delivery, RNA interference, PI3K–AKT–mTOR signaling

## Abstract

Intervertebral disc degeneration is a major cause of chronic low back pain, but effective disease-modifying therapies remain unavailable. Sustained and localized RNA interference (RNAi) offers a promising therapeutic approach; however, its application to the disc is limited by poor local retention and delivery-related toxicity. Here, we developed a release-controlled RNAi platform using biodegradable cationized gelatin nanospheres (cGNS) to deliver small interfering RNA (siRNA) targeting *Rptor*, which encodes RPTOR, a key component of the mechanistic target of rapamycin complex 1 (mTORC1). cGNS enabled longer-term intracellular siRNA retention with lower toxicity than conventional lipofection, leading to a prolonged decrease in RPTOR expression, autophagy activation, and suppressed apoptosis, senescence, and matrix breakdown. Intradiscal delivery of *Rptor* siRNA with cGNS preserved radiographic tissue morphology, maintained histological matrix integrity, and reduced cellular degeneration markers. Benefits were observed in both preventive and post-traumatic settings. cGNS-mediated controlled release of RNAi against *Rptor* is a durable therapeutic strategy for degenerative disc disease through mTORC1 inhibition.

## Highlights

• Cationized gelatin nanospheres (cGNS) are an siRNA delivery platform with safety.
• cGNS provide more efficient and sustained gene silencing than common lipofection.
• Knockdown of *Rptor*, encoding an mTORC1 component, maintains spinal disc health.
• Single-dose intradiscal *Rptor* RNAi by cGNS is potent for prevention and treatment.
• cGNS-mediated local mTORC1 inhibition is durable for degenerative disc disease.

## Graphical abstract

**Figure:**
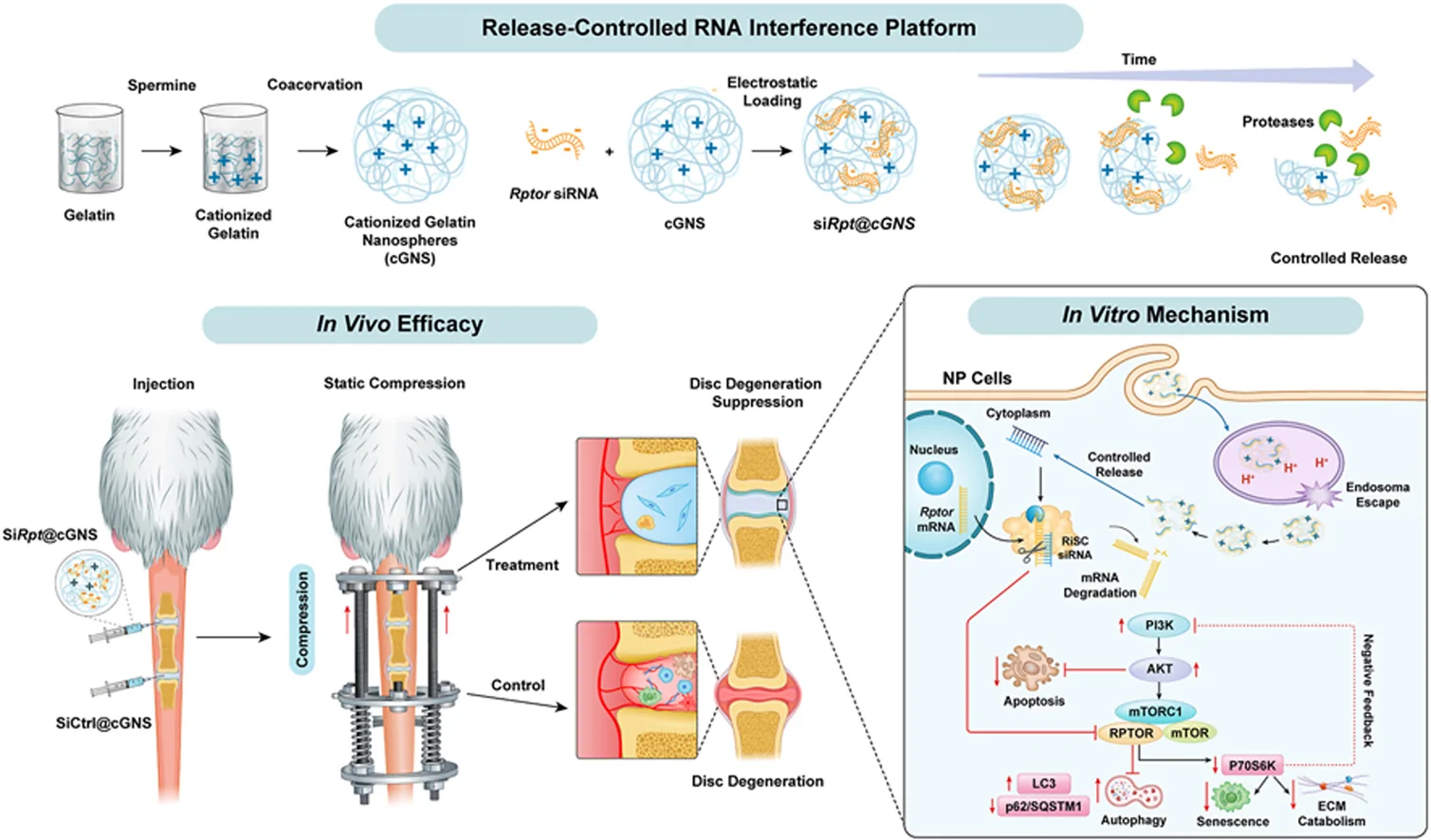

## 1 Introduction

Low back pain is a leading global cause of disability that profoundly affects the quality of life while imposing a considerable socioeconomic burden [[1], [2], [3], [4]]. Among its diverse etiologies, intervertebral disc degeneration is recognized as the principal contributor, particularly in the aging population [[5], [6], [7]]. Current conservative and surgical interventions, including anti-inflammatory drugs, physiotherapy, and spinal fusion, are largely palliative and do not restore disc structure or halt disease progression [5]. Therefore, there is an urgent need to develop biological strategies to slow or potentially reverse degeneration by directly targeting the underlying molecular mechanisms.

The intervertebral disc is a unique avascular tissue composed of the central nucleus pulposus (NP) surrounded by the annulus fibrosus (AF) and cartilaginous endplates [8]. The disc NP functions as a gel-like core that maintains compressive elasticity through its abundant extracellular matrix (ECM) of proteoglycans and collagen type II [9]. During aging, endplate calcification, nutrient deprivation, and mechanical stress disrupt disc NP cell homeostasis and initiate catabolic remodeling, apoptosis, and senescence [[10], [11], [12]]. These cellular events accelerate ECM degradation and compromise disc integrity, leading to chronic inflammation and tissue disruption [10,13,14]. In this context, anti-senescent biomaterials have recently been proposed as a promising strategy to disrupt the senescence-driven degenerative cascade in intervertebral disc degeneration [15].

At the molecular level, the phosphoinositide 3-kinase/protein kinase B/mechanistic target of rapamycin (PI3K–AKT–mTOR) signaling pathway integrates metabolic and stress cues to regulate cell growth, messenger RNA (mRNA) translation, protein synthesis, and autophagy [16]. Autophagy is a self-degradative process that removes and recycles damaged organelles and misfolded proteins and serves as an important homeostatic mechanism that enables cells to adapt to stressors such as hypoxia and nutrient deprivation [17]. The mTOR complex 1 (mTORC1), whose essential scaffolding component is the regulatory-associated protein of mTOR (RPTOR), plays a pivotal role in suppressing autophagy [16,[18], [19], [20]]. The mTOR signaling pathway is essential for cell growth and body metabolism [16,21,22]. However, overactivation of mTORC1 diminishes autophagic flux, resulting in the accumulation of damaged organelles, increased reactive oxygen species (ROS), and subsequent cell death and matrix catabolism [18,19,23,24]. Selective suppression of RPTOR using *RPTOR* small interfering RNA (siRNA) and pharmacological inhibition of mTORC1 using temsirolimus both restore autophagic activity and protect human disc NP cells from inflammation-induced apoptosis and senescence [25,26], indicating mTOR signaling as a key pathogenic driver of degeneration [19].

RNA interference (RNAi) provides an accurate and versatile approach for silencing disease-related genes such as *Rptor* [27,28]. Because RNAi acts in the cytoplasm without requiring nuclear entry or altering the genome, its effects are transient, diminish over successive cell divisions, and are not inherited by subsequent generations, supporting its potential safety as a therapeutic modality for prevalent, non-fatal diseases [24,29]. However, its therapeutic translation for intervertebral disc degeneration is limited by transient gene-silencing effects, rapid siRNA degradation, and inefficient cellular uptake in the dense hypoxic disc microenvironment [30]. Although siRNA-based therapeutics have entered clinical use, all currently approved formulations rely on systemic administration, and the development of drug delivery systems suitable for local application remains an unmet need [28,31]. Lipofection reagents, which are among the most commonly used transfection tools, enable transient *in vitro* delivery but lack the stability and compatibility required for *in vivo* use [32,33]. Moreover, ionizable lipid-based nanoparticles may induce local reactogenicity and innate immune activation, depending on their composition and route of administration [34]. Furthermore, repeated local injections into the disc can themselves cause degenerative injury through puncture. Thus, the ideal delivery system should provide superior and long-term efficiency after a single administration while minimizing invasiveness and maximizing safety [35,36]. Recent studies have highlighted the growing potential of regenerative biomaterials for spinal disorders, including anti-senescent biomaterials for intervertebral disc degeneration and scaffold-based strategies for spinal cord injury repair [37,38]. These advances further support the need for biomaterial-based local therapies tailored to the unique microenvironment of degenerative spinal tissues.

Cationized gelatin nanospheres (cGNS) have recently emerged as a promising biodegradable carrier for nucleic acid delivery [[39], [40], [41], [42], [43], [44], [45]]. Derived from safe gelatin modified with cationic amine groups, cGNS can electrostatically bind negatively charged siRNAs, enabling efficient encapsulation and gradual release [40,44]. Biocompatibility, enzymatic degradability, and charge-dependent binding properties of cGNS support controlled molecular retention, while markedly minimizing cytotoxicity [40,44]. We developed a controlled-release RNAi platform using cGNS for local delivery of *Rptor* siRNAs to intervertebral disc cells.

We hypothesized that *Rptor* siRNA (si*Rpt* in rats and si*RPT* in humans) incorporated into cGNS (si*Rpt*@cGNS, si*RPT*@cGNS) would sustain RPTOR suppression, inhibit mTORC1, enhance autophagy, and ultimately attenuate intervertebral disc degeneration. We further investigated whether cGNS could achieve sustained, biocompatible gene silencing *in vitro* and *in vivo*, thereby overcoming biological and technological limitations of conventional lipofection-mediated RNAi approaches (Fig. 1).

**Fig. 1:**
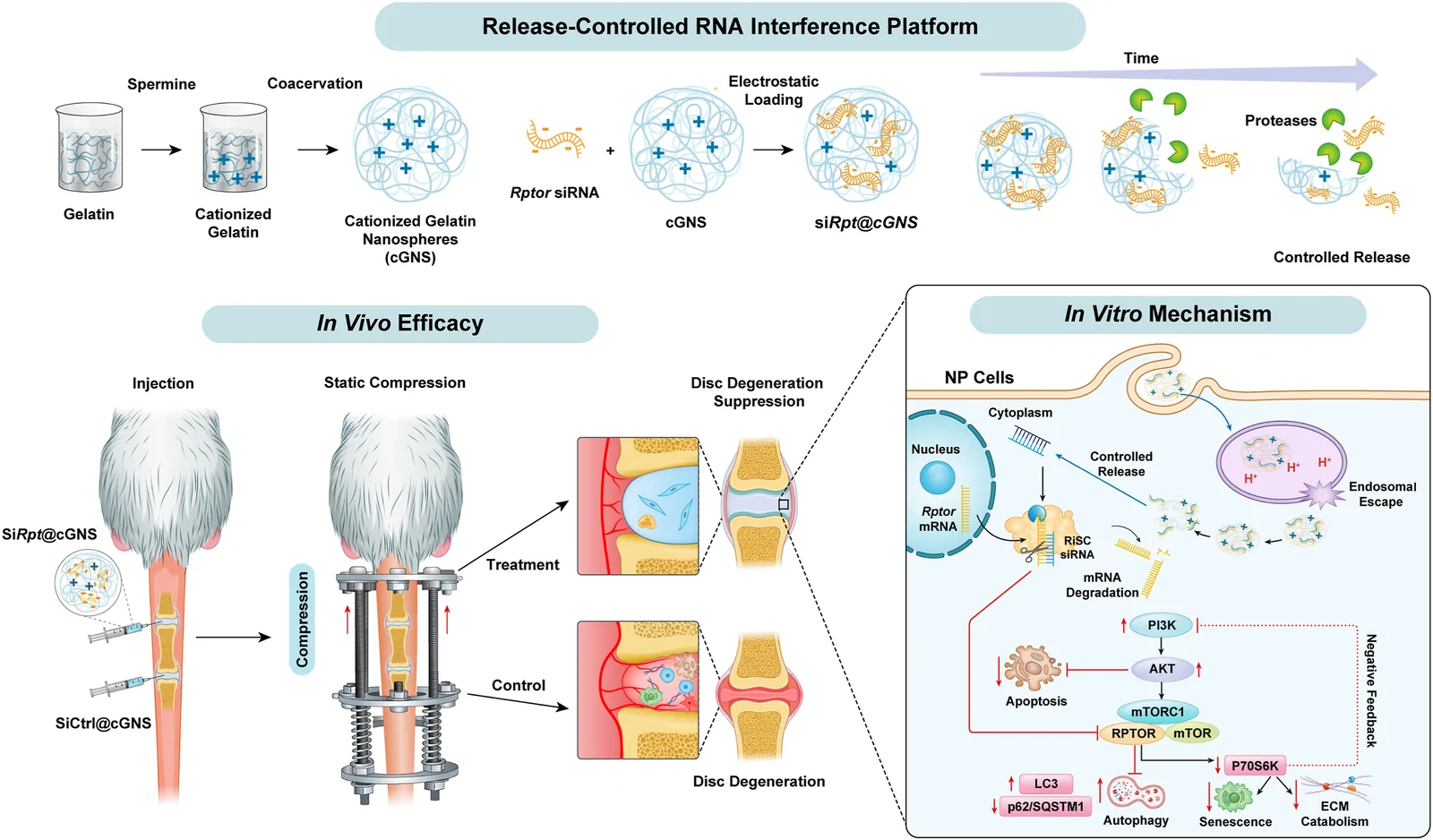
**Schematic overview of the release-controlled RNA interference platform targeting RPTOR and its protective mechanism against intervertebral disc degeneration**. Gelatin was first cationized with spermine and then processed by coacervation to generate cationized gelatin nanospheres (cGNS). *Rptor* siRNA was subsequently incorporated into the positively charged cGNS through electrostatic loading to form si*Rpt*@cGNS. Following the cellular uptake and endosomal escape, cGNS gradually degraded in a protease-responsive manner, enabling controlled intracellular release of siRNA over time. Released siRNA was incorporated into the RNA-induced silencing complex (RISC), resulting in degradation of *Rptor* mRNA and suppression of RPTOR expression. This sustained silencing modulated the PI3K–AKT–mTORC1 signaling axis, promoted autophagy, and suppressed apoptosis, senescence, and extracellular matrix (ECM) catabolism in disc NP cells. In the rat tail disc static compression model, intradiscal injection of si*Rpt*@cGNS attenuated structural disc degeneration compared with siCtrl@cGNS treatment, indicating the disc-protective potential of this release-controlled RNA interference platform.

## 2 Results

### 2.1 The mTORC1-associated transcriptional program is enriched in human intervertebral disc degeneration

To determine whether the transcriptional program associated with mTORC1 signaling is altered in human intervertebral disc degeneration, we reanalyzed five independent publicly available transcriptomic datasets (GSE23130, GSE165722, GSE244889, GSE199866, and GSE56081) comprising microarray, bulk RNA sequencing, and single-cell RNA sequencing datasets, with the single-cell data analyzed using a pseudobulk approach. In every dataset, degenerated discs were compared with less severely degenerated or non-degenerated controls, and each dataset was analyzed separately before the results were compared across the datasets.

Gene set enrichment analysis (GSEA) using the Molecular Signatures Database (MSigDB) Hallmark gene set HALLMARK_MTORC1_SIGNALING revealed a consistent, directionally concordant enrichment of the mTORC1-associated transcriptional program in intervertebral disc degeneration. The normalized enrichment score (NES) was positive in all five datasets (GSE23130, NES = 2.84; GSE165722, NES = 2.06; GSE199866, NES = 1.69; GSE56081, NES = 1.39; and GSE244889, NES = 1.14), reaching statistical significance (adjusted *p* < 0.05) in four of the five datasets (Fig. 2A). The single non-significant dataset, GSE244889, nonetheless showed enrichment in the same positive direction; thus, no dataset contradicted the overall pattern (Fig. S1).

**Fig. 2:**
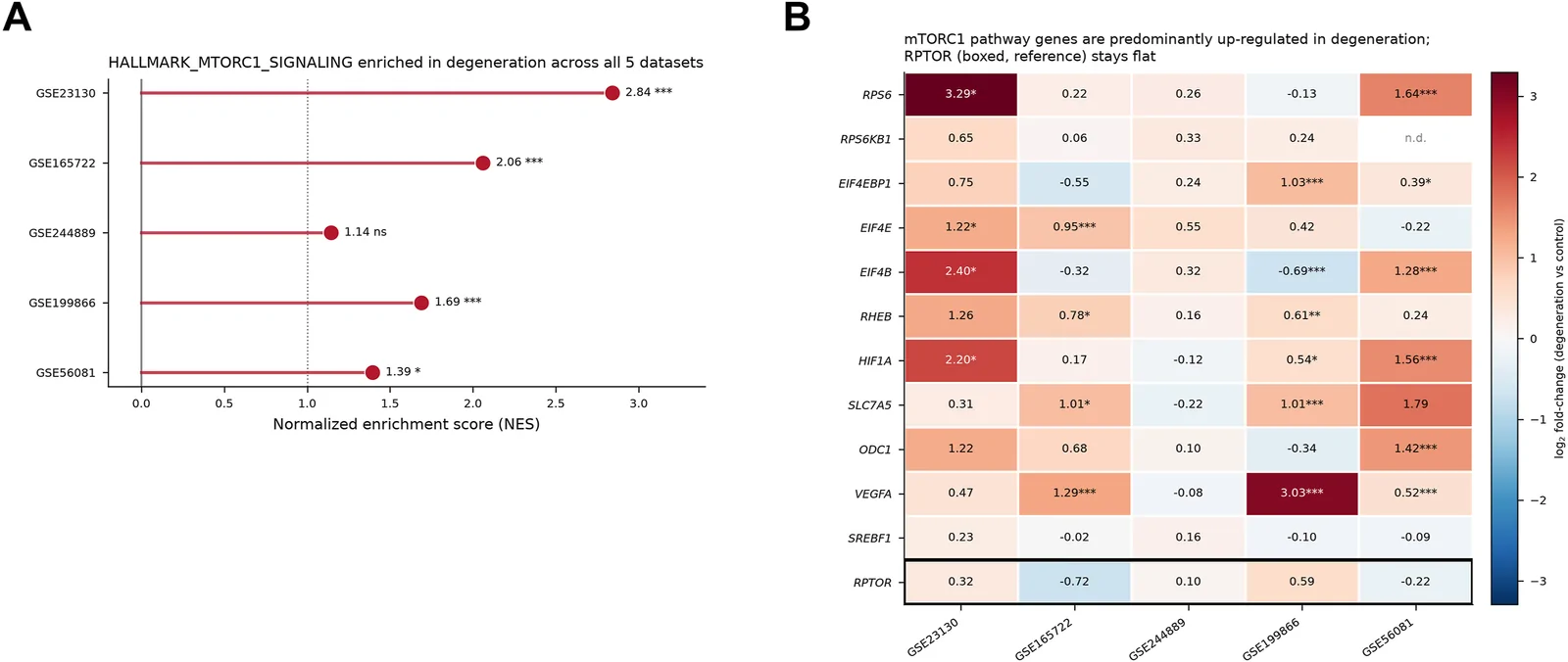
**The mTORC1-associated transcriptional program is consistently enriched across five human intervertebral disc degeneration datasets**. **A** Enrichment of the MSigDB Hallmark gene set HALLMARK_MTORC1_SIGNALING in degenerated versus control discs in each dataset, expressed as the NES from GSEA. Points indicate the NES. All five datasets showed positive enrichment, and significance based on Benjamini–Hochberg-adjusted *p* values is indicated (***adjusted *p* < 0.001, **adjusted *p* < 0.01, *adjusted *p* < 0.05, ns, not significant). **B** log_2_ fold changes in representative mTORC1-associated genes across the five datasets, including effector and target genes (*RPS6, RPS6KB1, EIF4EBP1, EIF4E, EIF4B, HIF1A, SLC7A5, ODC1, VEGFA,* and *SREBF1*) and the upstream activator *RHEB*. *RPTOR* (boxed row), encoding the mTORC1 subunit RAPTOR, was included as a reference and showed no consistent change. Cell color indicates the log_2_ fold change in degenerated versus control discs (red, upregulated; blue, downregulated). The log_2_ fold change and corresponding nominal *p* value are shown in each cell (***nominal *p* < 0.001, **nominal *p* < 0.01, *nominal *p* < 0.05); n.d., not detected. Datasets included GSE23130 (Affymetrix microarray), GSE165722 (single-cell RNA-seq, pseudobulk), GSE244889 (bulk RNA-seq), GSE199866 (single-cell RNA-seq, pseudobulk), and GSE56081 (Arraystar array with probe sequences reannotated to RefSeq mRNA). Each dataset was analyzed independently.

To relate this enrichment to individual pathway genes, we examined the behavior of an mTORC1 upstream regulator and a panel of representative mTORC1-associated genes across the five datasets. These mTORC1-associated genes, including the ribosomal protein S6 gene *RPS6*, the translation initiation factor genes *EIF4E* and *EIF4B*, the 4E-binding protein gene *EIF4EBP1*, and the mTORC1-associated metabolic and growth-related genes *HIF1A*, *SLC7A5*, *ODC1*, and *VEGFA*, were predominantly upregulated in degenerated discs, as was *RHEB*, which encodes an upstream activator of mTORC1. A minority of gene–dataset combinations showed no change or a modest decrease, consistent with the platform and cohort heterogeneity expected across independent studies; however, the dominant and reproducible signal was one of coordinated upregulation of mTORC1-associated genes (Fig. 2B).

In contrast to the pathway output genes, *RPTOR*, which encodes RAPTOR, the defining regulatory subunit of mTORC1, showed no consistent change across the five datasets and was included as a reference (boxed row, Fig. 2B). Its log_2_ fold changes ranged from −0.72 to +0.59, with none reaching statistical significance. These findings indicate that the observed enrichment reflects the transcriptional program associated with mTORC1 signaling rather than consistent transcriptional upregulation of *RPTOR* itself.

Overall, these results indicate that the mTORC1-associated transcriptional program is consistently enriched in human intervertebral disc degeneration across microarray, bulk RNA sequencing, and single-cell RNA sequencing platforms and also independent patient cohorts.

### 2.2 Design and synthesis of cGNS are optimized for sustained siRNA release

To develop a biodegradable nanocarrier for sustained intradiscal siRNA delivery, acid-processed porcine skin gelatin (PI9) was chemically modified by conjugation with spermine to introduce cationic amine groups (Fig. 3A). Quantification using the trinitrobenzene sulfonic acid (TNBS) assay demonstrated an amine substitution rate of 42.6 mol%, confirming substantial cationization of the gelatin backbone. Modified gelatin was recovered as a porous solid after lyophilization (Fig. 3B) and subsequently processed into nanospheres using a coacervation-based method.

**Fig. 3:**
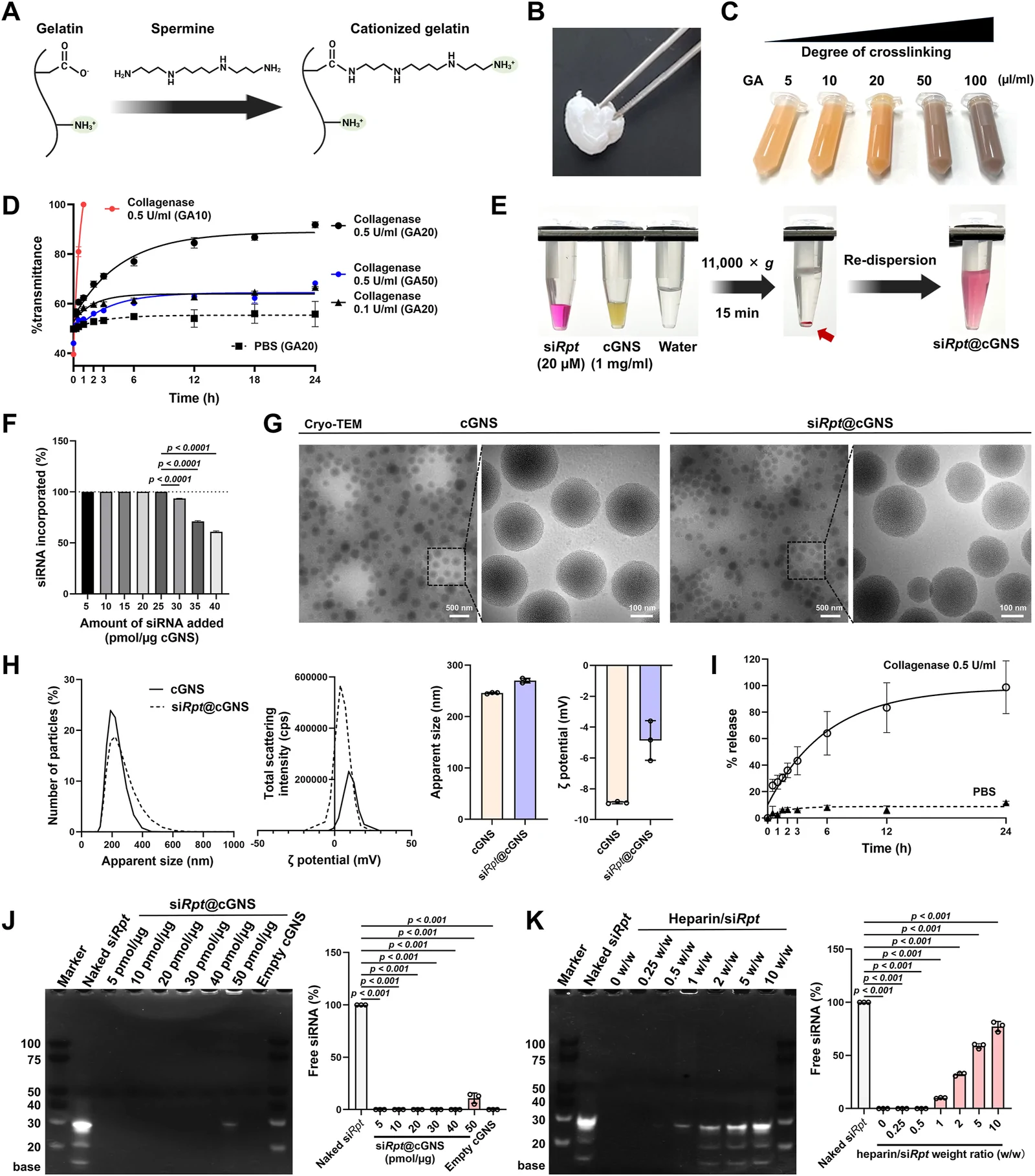
**Preparation and physicochemical characterization of *Rptor* siRNA (si*Rpt*) incorporated into cationized gelatin nanospheres (cGNS) (si*****Rpt*****@cGNS)**. **A** Chemical structures of PI9 gelatin, spermine, and cationized gelatin. Cationization introduced abundant amine (–NH_2_) groups for electrostatic interaction with siRNA. **B** Macroscopic appearance of lyophilized cationized gelatin. **C** cGNS were generated using increasing glutaraldehyde (GA) concentrations (5–100 μL/mL). **D** Enzymatic degradation profiles of cGNS with different crosslinking densities in collagenase-containing solution and PBS (*n* = 3 independent experiments, mean ± s.d.). **E** Schematic illustration of si*Rpt*@cGNS preparation via centrifugation-mediated siRNA incorporation. **F** siRNA loading capacity of cGNS. Incorporation reached saturation above 25 pmol siRNA per μg cGNS (*n* = 3 independent experiments, mean ± s.d.). **G** Cryo-TEM images of cGNS and si*Rpt*@cGNS. Scale bars, 1 μm and 100 nm. **H** Size and zeta-potential distributions of cGNS and si*Rpt*@cGNS, with quantification of apparent size and zeta potential (*n* = 3 independent experiments, mean ± s.d.). **I***In vitro* release profiles of siRNA from si*Rpt*@cGNS in PBS or collagenase-containing solution (*n* = 3 independent experiments, mean ± s.d.). **J** Gel-retardation assay of si*Rpt*@cGNS prepared at increasing siRNA-to-cGNS loading ratios, including 5, 10, 15, 20, 25, and 30 pmol siRNA per μg cGNS, with quantification of freely migrating siRNA (*n* = 3 independent experiments, mean ± s.d.). **K** Heparin displacement assay of si*Rpt*@cGNS at increasing heparin-to-siRNA weight ratios, with quantification of displaced free siRNA (*n* = 3 independent experiments, mean ± s.d.). Statistical significance in **F**, **J**, and **K** was determined using one-way ANOVA followed by Dunnett's multiple comparison test.

To regulate structural stability and degradation behavior, glutaraldehyde (GA) crosslinking densities were systematically optimized. GA was added at 5–100 μL per 1 mL of gelatin solution, generating nanospheres with distinct degrees of crosslinking (Fig. 3C). As degenerative intervertebral discs are characterized by elevated collagenase activity, enzyme-responsive degradation was evaluated. cGNS maintained structural integrity in phosphate-buffered saline (PBS) but underwent collagenase concentration-dependent degradation. Among the tested formulations, GA at 20 μL/mL exhibited an intermediate degradation profile, with neither excessively rapid nor overly delayed degradation (Fig. 3D). Therefore, GA concentration of 20 μL/mL was selected for subsequent experiments to balance particle stability and enzyme-responsive degradability, consistent with previous reports [40,43,44].

*Rptor* siRNA was complexed with cGNS via centrifugation-assisted mixing to generate si*Rpt*@cGNS (Fig. 3E). Loading efficiency analysis demonstrated that siRNA incorporation reached saturation at approximately 30 pmol/μg cGNS. Incorporation at 20 pmol/μg cGNS achieved near-maximal loading while minimizing unbound siRNA; therefore, this ratio was selected as the standard siRNA/cGNS formulation for subsequent experiments (Fig. 3F). Thus, the formulation condition was determined based on the maximum loading capacity of cGNS rather than by arbitrary dose selection.

Cryo-transmission electron microscopy (TEM) revealed that cGNS and si*Rpt*@cGNS consisted of discrete, nearly spherical nanoparticles with diameters on the order of 200 nm. The particles showed relatively uniform morphology without large evident aggregates. No marked differences in gross morphology were observed between cGNS and si*Rpt*@cGNS, indicating that siRNA incorporation did not substantially alter the nanospherical morphology of cGNS (Fig. 3G and **Supplementary Movie A**). Dynamic light scattering analysis showed that blank cGNS had a hydrodynamic diameter of 245.8 ± 1.4 nm, low polydispersity index (PdI) of 0.015 ± 0.052, and positively charged surface of +8.90 ± 0.07 mV (Fig. 3H). Following siRNA incorporation, the particle size of cGNS increased modestly to 270.0 ± 4.4 nm, whereas the zeta potential decreased to +4.87 ± 1.29 mV, consistent with siRNA complexation. Then, PdI remained low at 0.074 ± 0.008, still indicating a narrow particle-size distribution without substantial aggregation (Fig. 3H). Fluorescently labeled derivatives showed comparable size and surface charge characteristics, indicating that labeling preserved the structural properties of particles (Table S1).

Consistent with the degradation profile, siRNA release from 20 μL/mL GA-mediated cGNS was negligible in PBS but markedly accelerated under collagenase-containing conditions (Fig. 3I). Release kinetic analysis showed that si*Rpt*@cGNS in collagenase-containing buffer exhibited an initial burst release of 24.7% ± 4.6% at 0.5 h and reached near-complete release at 24 h (98.8% ± 19.9%), with a fitted plateau of approximately 103% and an estimated half-release time of 4.1 h. In contrast, release in PBS remained limited at 24 h (11.6% ± 1.1%) without reaching a saturation plateau (Table S2).

In the gel retardation assay, naked siRNA migrated into the gel, whereas siRNA incorporated into cGNS was predominantly retained in the loading wells, indicating markedly reduced electrophoretic mobility following complex formation. Free siRNA remained nearly undetectable at lower siRNA-to-cGNS ratios but gradually appeared at higher ratios, suggesting the saturation of the siRNA-loading capacity of cGNS (Fig. 3J). In the heparin displacement assay, free siRNA was undetectable or minimally detected in the absence of heparin and at low heparin/siRNA weight ratios of 0.25 and 0.5. At ratios of 1 or higher, the free siRNA band gradually increased with heparin concentration, reaching approximately 80% of the naked siRNA control at a weight ratio of 10 (Fig. 3K).

These results indicate that cGNS support degradation-dependent siRNA release under enzyme-rich conditions and are suitable for sustained RNAi in the intradiscal microenvironment. Together, these sequential optimization experiments established 20 μL/mL GA-crosslinked cGNS loaded with 20 pmol siRNA per μg cGNS as the standard si*Rpt*@cGNS formulation, whereas 400 pmol/mL siRNA-equivalent si*Rpt*@cGNS was selected as the maximum non-cytotoxic working concentration for functional *in vitro* experiments (Fig. 6A).

### 2.3 *In vitro* si*Rpt*@cGNS facilitates sustained cellular uptake, intracellular trafficking, endosomal escape, and autophagy activation

To ensure physiological relevance, NP cells were isolated from coccygeal (Co) intervertebral discs of 12-week-old male Sprague–Dawley rats. Western blotting (WB) confirmed that early passage cells retained disc NP notochordal characteristics, including brachyury and CD24 expression [8], and only first-passage and second-passage cells were used (Fig. S2). To minimize potential off-target effects, three independent *Rptor* siRNA sequences were screened (Table S3). In WB, sequence 2 achieved the most robust reduction in RPTOR expression and was selected for all subsequent experiments (Fig. S3).

Next, we assessed cellular uptake and intracellular retention of fluorescently labeled cGNS and siRNA following si*Rpt*@cGNS administration. At 24 h, FITC-labeled cGNS and Cy5-labeled *Rptor* siRNA exhibited clear cytoplasmic colocalization, indicating efficient internalization (Fig. 4A). Quantitative analysis revealed sustained intracellular signals over time (Fig. 4B). cGNS and siRNA signals remained detectable for up to 14 days, including after one passage (Fig. 4A and B). Moreover, the signals were distributed relatively evenly between daughter cells after division (Fig. 4C). To further evaluate intracellular retention after cell division, we examined cGNS and siRNA signals during serial passaging. Both signals remained detectable after repeated passages, with merged signals observed up to 14 days (P + 3) after transfection (Fig. 4D). These findings demonstrate a prolonged intracellular retention and partitioning of the labeled material across multiple cell divisions rather than rapid clearance.

**Fig. 4:**
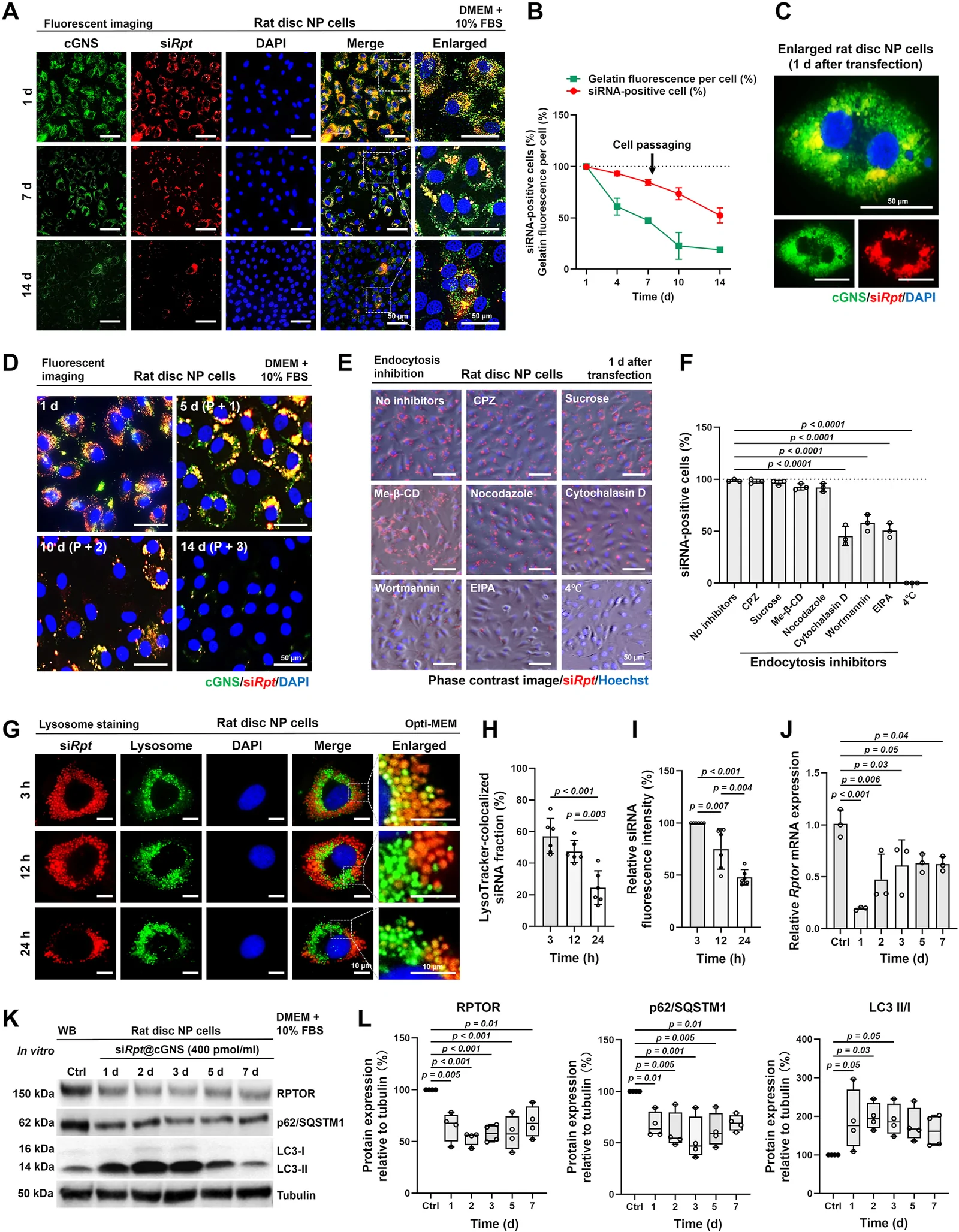
**Cellular uptake, intracellular retention, trafficking, and autophagy induction by si*Rpt*@cGNS in rat disc NP cells**. **A** Fluorescence images showing uptake and intracellular retention of si*Rpt*@cGNS in rat NP cells. cGNS were labeled with FITC (green), and *Rptor* siRNA was labeled with Cy5 (red). Cells were passaged 7 days after treatment. Scale bars, 50 μm. **B** Time-course analysis of intracellular cGNS fluorescence intensity and the percentage of siRNA-positive cells shown in **A** (*n* = 3 biologically independent experiments, mean ± s.d.). **C** Distribution of cGNS and siRNA during NP cell division, showing cytoplasmic persistence after mitosis. Scale bar, 50 μm. **D** Intracellular retention of cGNS and siRNA after serial passaging up to passage P+3. Scale bars, 50 μm. **E** Cellular uptake of si*Rpt*@cGNS in NP cells after treatment with endocytosis inhibitors. Scale bars, 50 μm. **F** Quantification of siRNA-positive cells shown in **E** (*n* = 3 biologically independent experiments, mean ± s.d.). **G** Intracellular localization of Cy5-labeled si*Rpt* and lysosomes at 3, 12, and 24 h. Scale bars, 10 μm. **H** Quantification of the fraction of siRNA colocalized with lysosomes using Manders' tM1 (*n* = 6 biologically independent experiments, mean ± s.d.). **I** Relative siRNA fluorescence intensity at 3, 12, and 24 h (*n* = 6 biologically independent experiments, mean ± s.d.). **J** Time-course analysis of relative *Rptor* mRNA expression following si*Rpt*@cGNS treatment (*n* = 3 biologically independent experiments, mean ± s.d.). **K** WB of RPTOR and autophagy-related proteins following si*Rpt*@cGNS treatment. **L** Quantification of RPTOR, p62/SQSTM1, and the LC3-II/I ratio shown in **K** (*n* = 4 biologically independent experiments, mean ± s.d.). Statistical significance in **F**, **J**, and **L** was determined using one-way ANOVA followed by Dunnett's multiple comparison test. Statistical significance in **H** and **I** was determined using one-way ANOVA followed by Tukey's multiple comparison test.

To further assess translational applicability, we performed pilot validation experiments using primary human degenerative disc NP cells isolated from surgical specimens obtained from patients of different ages and with different degeneration grades (Fig. S4A). Fluorescently labeled siRNA incorporated into cGNS showed an efficient intracellular uptake in human primary degenerative disc NP cells (Fig. S4B). Moreover, cGNS incorporating human-specific *RPTOR* siRNA reduced RPTOR protein expression, suggesting that cGNS-mediated RNAi is applicable to human degenerative disc NP cells as well (Fig. S4C).

To identify the endocytic pathway involved in cGNS internalization, rat disc NP cells were pretreated with specific inhibitors. Inhibitors of clathrin-mediated and caveolae-mediated endocytosis, including chlorpromazine, sucrose, and methyl-β-cyclodextrin, did not significantly reduce uptake. In contrast, nocodazole, cytochalasin D, wortmannin, and 5-(N-ethyl-N-isopropyl) amiloride markedly suppressed internalization (Fig. 4E and F). Macropinocytosis is thus a major uptake route for cGNS in disc NP cells.

Following internalization, functional siRNA activity requires escape from endolysosomal compartments. To characterize intracellular trafficking of the siRNA cargo and cGNS carrier, lysosomal colocalization and intracellular fluorescence intensity were evaluated over 24 h after si*Rpt*@cGNS administration. Colocalization of Cy5-labeled *Rptor* siRNA with lysosomes progressively decreased from 3 h and was markedly reduced at 24 h (Fig. 4G and H), accompanied by a gradual decline in intracellular siRNA fluorescence intensity (Fig. 4I). Conversely, in a consecutive observation, Cy5-labeled cGNS showed more extensive lysosomal colocalization than siRNA at 3 h, which subsequently decreased and was markedly reduced at 24 h (Fig. S5A and B). Intracellular cGNS fluorescence intensity also declined over the 24-h period (Fig. S5C). These distinct temporal patterns suggest an earlier dissociation of siRNA from lysosomal compartments, whereas cGNS initially remain more strongly colocalized with lysosomes before subsequently redistributing.

To confirm increased autophagic flux induced by RPTOR suppression rather than static autophagosome accumulation, rat disc NP cells were first transfected with *Rptor* siRNA through lipofection as a mechanistic control. mTORC1 inhibition through reduced RPTOR expression significantly increased LC3 puncta formation, and co-treatment with bafilomycin A1 (BafA1), a lysosomal inhibitor [46], further increased LC3 puncta, indicating the activation of autophagic flux (Fig. S6A and B).

Finally, we investigated whether si*Rpt*@cGNS induced sustained RNAi-mediated *Rptor* knockdown and autophagy activation. Quantitative reverse transcription PCR (RT–qPCR) analysis first showed that *Rptor* mRNA expression was markedly reduced as early as 1 day after si*Rpt*@cGNS treatment, and this suppression was maintained up to 7 days, although the knockdown effect gradually attenuated over time (Fig. 4J). Consistent with this transcript-level silencing, WB demonstrated a sustained reduction in RPTOR protein expression for up to 7 days, accompanied by a persistent increase in the LC3-II/LC3-I ratio and decrease in p62/SQSTM1 expression (Fig. 4K,L). These findings indicate that si*Rpt*@cGNS efficiently enters disc NP cells, undergoes endolysosomal escape, and mediates sustained *Rptor* knockdown together with prolonged autophagy activation.

### 2.4 *In vitro* si*Rpt*@cGNS-mediated RPTOR suppression rewires PI3K–AKT–mTOR signaling and promotes a survival-adaptive phenotype

To examine how selective mTORC1 inhibition affects downstream signaling and cellular responses, we first outlined the anticipated consequences of si*Rpt*@cGNS-mediated suppression of RPTOR, which is a core component of mTORC1 (Fig. 5A). We then examined the effects of *Rptor* knockdown on phosphorylation signaling and stress responses in rat disc NP cells. Reduced RPTOR expression markedly suppressed the phosphorylation of p70S6K, which is a canonical downstream effector of mTORC1 [47] (Fig. 5B–D). Consistent with mTORC1 feedback regulation [47], p70S6K suppression was accompanied by increased AKT phosphorylation (Fig. 5B–D). RPTOR suppression also significantly reduced ULK1 phosphorylation at Ser757 without altering total ULK1 expression, providing a direct mechanistic link between mTORC1 inhibition and autophagy induction (Fig. 5B–D). Moreover, BafA1 further increased LC3 puncta accumulation in *Rptor*-silenced cells, supporting enhanced autophagic flux (Fig. S6A and B). In contrast, the phosphorylation ratios of AMPKα and GSK3β were not significantly altered (Fig. S7A and B), indicating that autophagy activation is predominantly associated with the mTORC1–ULK1 axis rather than broad activation of multiple stress-response pathways.

**Fig. 5:**
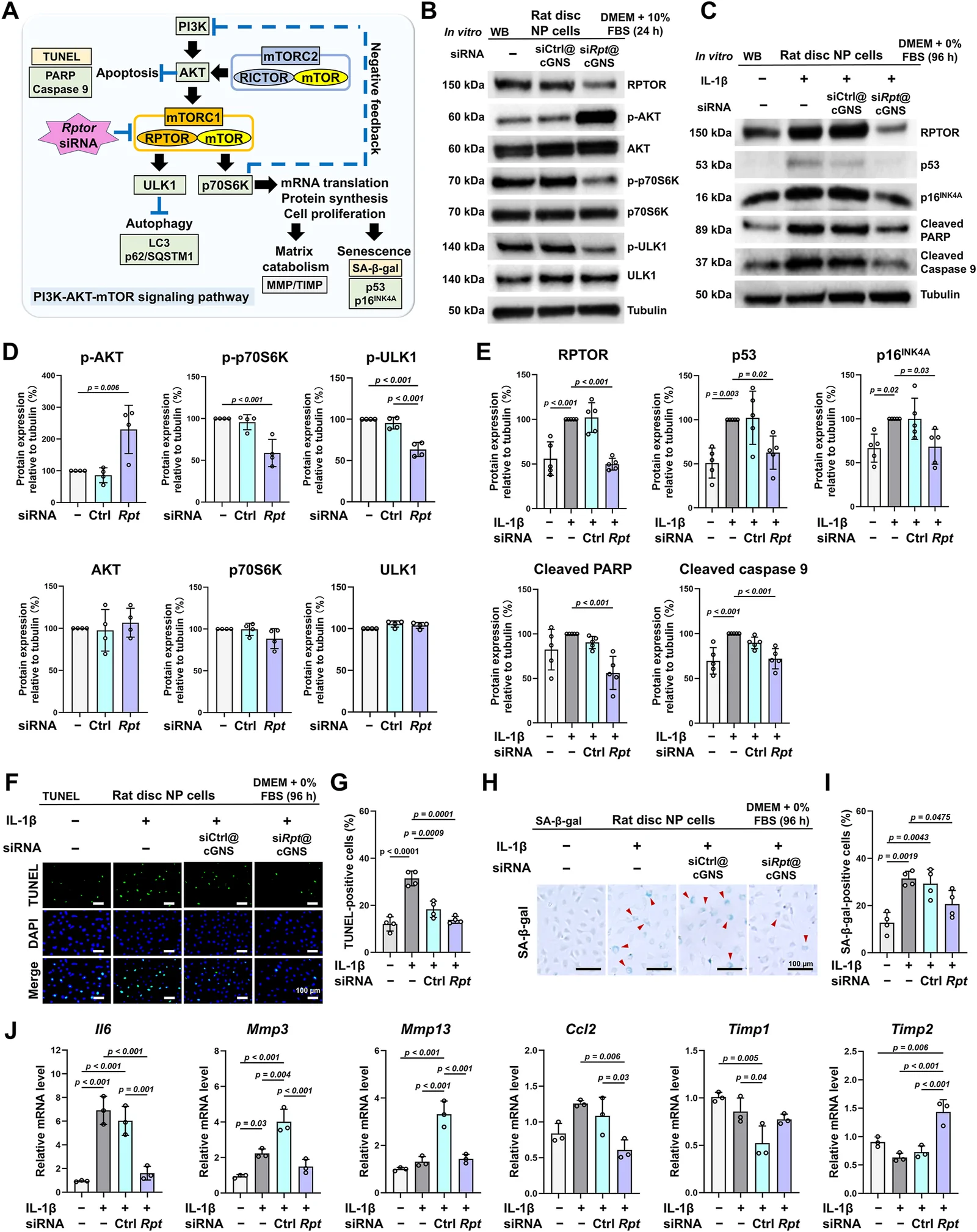
**Effects of *Rptor* silencing on PI3K–AKT–mTOR signaling, inflammation-induced apoptosis, and senescence in rat disc NP cells**. **A** Schematic illustration of the PI3K–AKT–mTOR signaling pathway, highlighting the regulatory role of RPTOR and the expected consequences of RPTOR suppression. **B** WB of RPTOR, p-AKT (Ser473), AKT, p-p70S6K (Thr389), p70S6K, p-ULK1 (Ser757), and ULK1 in rat NP cells treated with siCtrl@cGNS or si*Rpt*@cGNS. **C** WB of apoptosis- and senescence-associated proteins in rat NP cells treated with siCtrl@cGNS or si*Rpt*@cGNS under pro-inflammatory conditions induced by IL-1β. **D** Quantification of phosphorylated and total AKT, p70S6K, and ULK1 shown in **B** (*n* = 4 biologically independent experiments, mean ± s.d.). **E** Quantification of RPTOR and apoptosis- and senescence-related proteins shown in **C** (*n* = 5 biologically independent experiments, mean ± s.d.). **F** TUNEL staining of NP cells treated with siCtrl@cGNS or si*Rpt*@cGNS under inflammatory conditions. Scale bar, 50 μm. **G** Quantification of TUNEL-positive cells shown in **F** (*n* = 4 biologically independent experiments, mean ± s.d.). **H** SA-β-gal staining of NP cells treated with siCtrl@cGNS or si*Rpt*@cGNS under inflammatory conditions. Scale bar, 100 μm. **I** Quantification of SA-β-gal-positive cells shown in **H** (*n* = 4 biologically independent experiments, mean ± s.d.). **J** RT-qPCR analysis of inflammation- and ECM-related gene expression, including *Il6*, *Mmp3*, *Mmp13*, *Ccl2*, *Timp1*, and *Timp2*, in NP cells treated with siCtrl@cGNS or si*Rpt*@cGNS under inflammatory conditions (*n* = 3 biologically independent experiments, each performed in duplicate, mean ± s.d.). Statistical significance was determined using one-way ANOVA followed by Tukey's multiple comparison test.

These signaling changes were accompanied by cytoprotective effects. Upon pro-inflammatory IL-1β stimulation, si*Rpt*@cGNS-mediated RPTOR suppression significantly reduced apoptotic cleavage of PARP and caspase 9 and decreased the expression of senescence markers p53 and p16^INK4A^ (Fig. 5C–E). Pro-apoptotic protein BAX was also reduced, whereas anti-apoptotic BCL2 expression was not affected (Fig. S7A and B). Quantitative assessments further confirmed significant reductions in apoptotic terminal deoxynucleotidyl transferase dUTP nick end labeling (TUNEL)-positive cells (Fig. 5F and G) and senescence-associated β-galactosidase (SA-β-gal)-positive cells (Fig. 5H and I).

We further assessed ECM metabolism and senescence-associated secretory phenotype (SASP)-related gene expression, both of which are closely related to disc degeneration. While IL-1β significantly elevated pro-inflammatory *Il6* and catabolic *Mmp3* expression, si*Rpt*@cGNS downregulated *Il6*, *Mmp3*, *Mmp13*, and a SASP factor *Ccl2* but concomitantly upregulated anti-catabolic *Timp1* and *Timp2* compared with siCtrl@cGNS (Fig. 5J). In addition, si*Rpt*@cGNS reduced the expression of SASP factors *Pai1* and *Cxcl1* under IL-1β stimulation (Fig. S7C).

To exclude non-specific cytotoxic effects, water-soluble tetrazolium-8 (WST-8) assay was first performed using control, naked siRNA, cGNS alone, siCtrl@cGNS, and si*Rpt*@cGNS groups. No marked differences in cell viability were observed among the groups over the tested time points, indicating that cGNS alone, naked siRNA, and siCtrl@cGNS did not substantially affect NP cell viability under these conditions (Fig. S8A). Similarly, 2′,7′-dichlorodihydrofluorescein diacetate (DCFH-DA) assay showed no significant increase in intracellular ROS after treatment with naked siRNA, cGNS alone, siCtrl@cGNS, or si*Rpt*@cGNS, whereas H_2_O_2_ induced a marked increase as a positive control (Fig. S8B and C). In addition, live–dead assay was performed. Neither siCtrl@cGNS nor si*Rpt*@cGNS induced significant cytotoxicity at the tested concentrations (Fig. S9A and B), and the total cell number was comparable between the groups (Fig. S9C). Despite mTORC1 inhibition, 5-ethynyl-2′-deoxyuridine (EdU) incorporation significantly increased in si*Rpt*@cGNS-treated cells, indicating maintained proliferative capacity (Fig. S9D and E). Together, these findings indicate that si*Rpt*@cGNS-mediated RPTOR suppression inhibits mTORC1–p70S6K signaling, reduces inhibitory ULK1 Ser757 phosphorylation, and enhances autophagic flux, further attenuating apoptosis, senescence, and ECM catabolism. Concomitant increase in AKT phosphorylation is consistent with compensatory feedback activation, although its functional contribution to the protective effects remains to be determined.

### 2.5 *In vitro* cGNS enable high-dose, low-toxicity siRNA delivery with sustained RPTOR suppression

To evaluate translational advantages of cGNS as a sustained and biocompatible siRNA delivery platform, we compared si*Rpt*@cGNS with a conventional lipofection-based reagent (si*Rpt*@Lipo). WST-8 assay revealed a marked difference in disc cell viability, as si*Rpt*@cGNS permitted siRNA delivery at concentrations up to 400 pmol/mL without detectable cytotoxicity, while si*Rpt*@Lipo induced significant toxicity at concentrations above 60 pmol/mL (Fig. 6A). Consistent with the morphological analysis, si*Rpt*@Lipo-treated cells exhibited a pronounced cell shrinkage and loss of normal morphology at 400 pmol/mL, whereas si*Rpt*@cGNS-treated cells maintained a healthy appearance under the same condition (Fig. 6B).

**Fig. 6:**
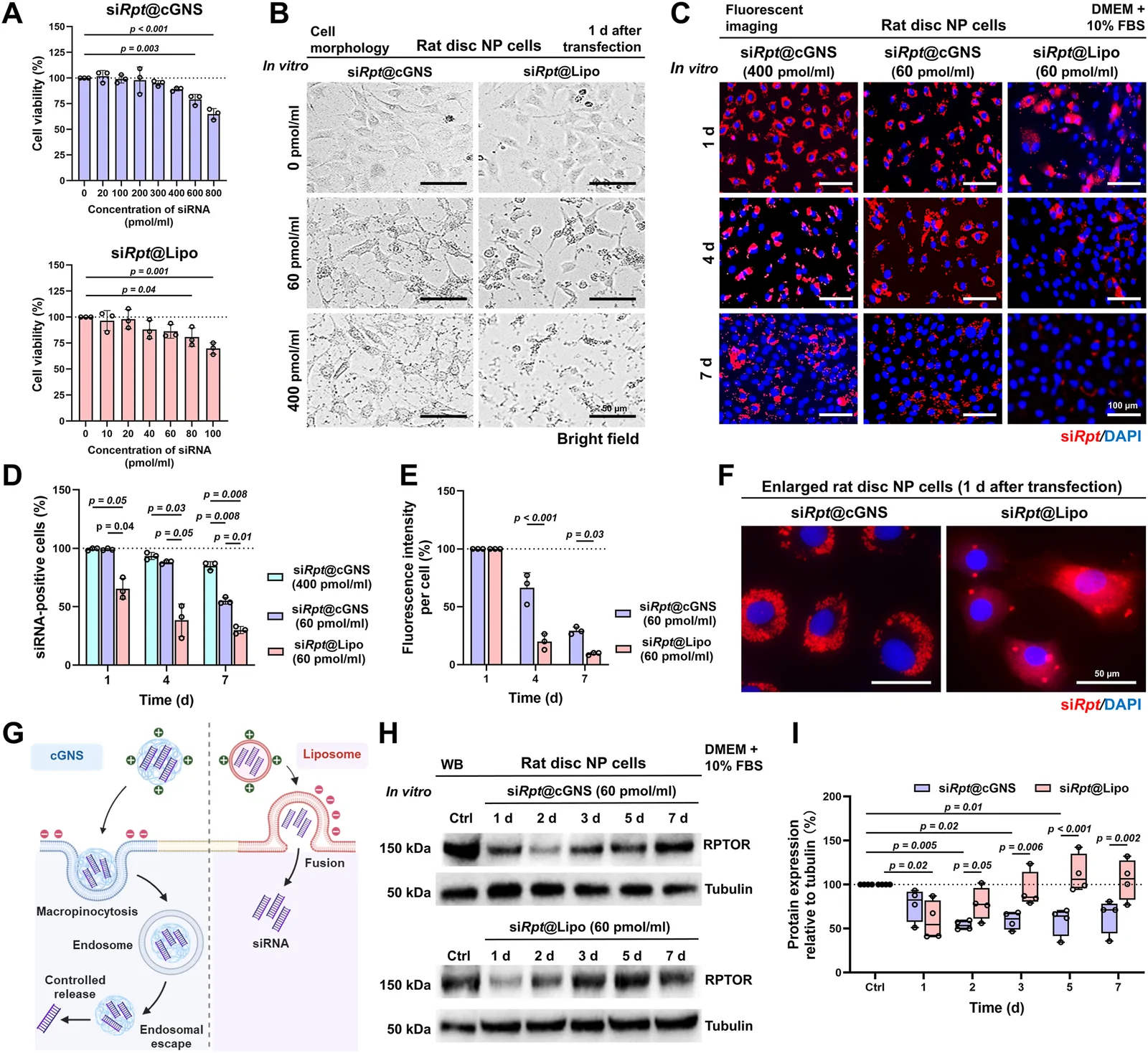
**Comparison of cytocompatibility, intracellular retention, and knockdown efficiency between si*Rpt*@cGNS and si*****Rpt*****@Lipo in rat disc NP cells**. **A** Cytotoxicity analysis of si*Rpt*@cGNS and si*Rpt*@Lipo in rat NP cells across increasing siRNA concentrations (*n* = 3 biologically independent experiments, mean ± s.d.). **B** Morphological assessment of NP cells treated with increasing concentrations of siRNA delivered using si*Rpt*@cGNS or si*Rpt*@Lipo. Scale bar, 50 μm. **C** Fluorescence images showing intracellular retention of Cy5-labeled siRNA in NP cells after treatment with si*Rpt*@cGNS or si*Rpt*@Lipo. Scale bar, 100 μm. **D** Quantification of the percentage of siRNA-positive cells across treatment groups and time points shown in **C** (*n* = 3 biologically independent experiments, mean ± s.d.). **E** Quantification of intracellular Cy5 fluorescence intensity per cell shown in **C**, normalized to the corresponding value on Day 1 (*n* = 3 biologically independent experiments, mean ± s.d.). **F** High-magnification images illustrating differences in intracellular siRNA distribution between si*Rpt*@cGNS and si*Rpt*@Lipo. Scale bar, 50 μm. **G** Schematic depiction of distinct cytoplasmic uptake and trafficking pathways mediated by cGNS- and liposome-based delivery systems. **H** WB comparing RPTOR suppression following treatment with si*Rpt*@cGNS or si*Rpt*@Lipo using equivalent siRNA doses. **I** Quantification of RPTOR protein levels shown in **H** across treatment groups and time points (*n* = 4 biologically independent experiments, mean ± s.d.). Statistical significance in **A** was determined using one-way ANOVA followed by Dunnett's multiple comparison test. Data in **D**, **E**, and **I** were analyzed using two-way ANOVA followed by Tukey's multiple comparison test.

Subsequently, we compared intracellular retention of fluorescently labeled siRNA between the two delivery systems. si*Rpt*@cGNS was evaluated at the maximum non-toxic concentration (400 pmol/mL) and at a concentration equivalent to that used for lipofection (60 pmol/mL). At both concentrations, si*Rpt*@cGNS-treated cells exhibited a greater initial intracellular fluorescence and more prolonged signal persistence than si*Rpt*@Lipo-treated cells (Fig. 6C and D). To compare signal retention independently of baseline fluorescence intensity, signals at each time point were normalized to the Day 1 value within each group. After normalization, si*Rpt*@cGNS-treated cells retained a greater proportion of the initial intracellular fluorescence over time than si*Rpt*@Lipo-treated cells (Fig. 6E).

High-magnification imaging showed distinct intracellular distribution patterns. While si*Rpt*@cGNS generated discrete punctate cytoplasmic signals, si*Rpt*@Lipo-treated cells displayed a combination of punctate and diffuse cytoplasmic signals (Fig. 6F), which is consistent with the difference in intracellular handling between the two systems (Fig. 6G).

WB using equivalent siRNA doses demonstrated that si*Rpt*@cGNS significantly reduced RPTOR protein expression from Day 2 onward and maintained suppression throughout the observation period, whereas si*Rpt*@Lipo induced a robust early suppression that declined rapidly (Fig. 6H and I). Thus, cGNS enable higher-dose siRNA delivery with lower cytotoxicity and more sustained RPTOR suppression than conventional lipofection.

### 2.6 *In vivo* cGNS provide prolonged intradiscal retention and NP-preferential localization of labeled siRNA

To establish an anatomically appropriate intradiscal delivery protocol, we first determined the maximum injectable volume that could be retained within the disc NP compartment. Intradiscal injection with varying volumes was performed using a 33-gauge (33G) needle to minimize tissue disruption [48] (Fig. 7A). Radiography and micro-computed tomography (CT) discography revealed that injection volumes exceeding 2 μL resulted in substantial leakage from the NP; therefore, 2 μL was selected as the optimal injection volume for subsequent experiments (Fig. S10A). Preliminary dose-optimization experiments showed that 60 pmol/μL siRNA incorporated into 3 μg/μL cGNS was required to achieve uniform NP distribution without peripheral leakage (Fig. S10B). In this formulation, si*Rpt*@cGNS was a flowable aqueous suspension and could be readily delivered through a 33G needle, enabling successful injection into the intervertebral disc NP region (Fig. S10C and D and **Supplementary Movie B**). Additionally, passage through a 33G needle did not substantially alter the apparent particle size, PdI, or zeta potential of si*Rpt*@cGNS (apparent size, 255.8 ± 1.9 to 239.0 ± 1.6 nm; PdI, 0.031 ± 0.057 to 0.024 ± 0.069; zeta potential, 5.06 ± 0.51 to 5.20 ± 0.80 mV, before and after passage, respectively).

**Fig. 7:**
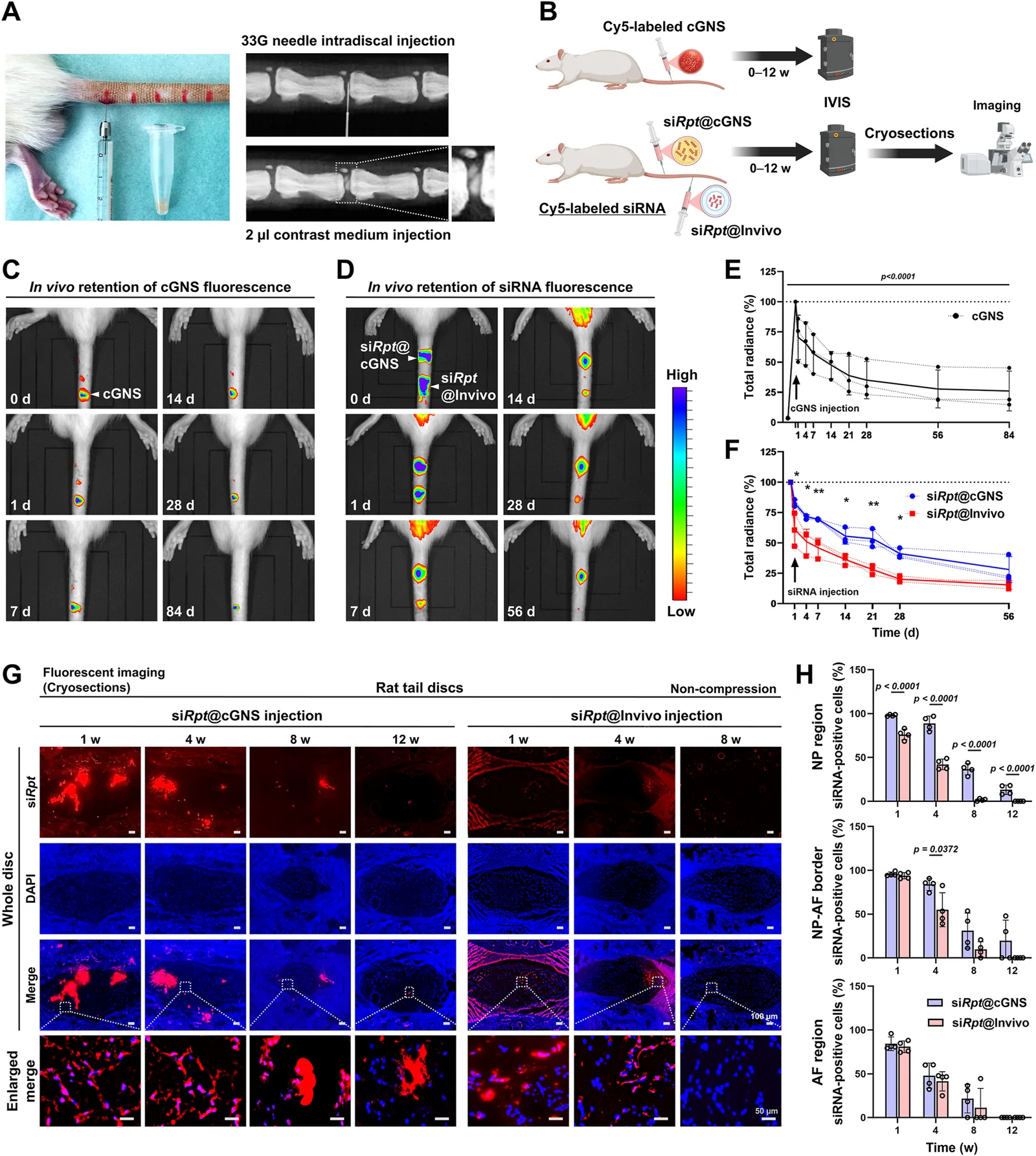
***In vivo* degradation, release kinetics, and intradiscal retention of siRNA delivered by si*****Rpt*****@cGNS**. **A** Schematic illustration of intradiscal injection into rat tail intervertebral discs under fluoroscopic guidance using a 33G needle. A volume of 2 μL per disc was injected. **B** Experimental overview of *in vivo* cGNS degradation, siRNA release, and intracellular uptake using fluorescently labeled cGNS and siRNA. **C** IVIS imaging showing longitudinal *in vivo* degradation of fluorescently labeled cGNS following intradiscal injection. **D** IVIS imaging showing *in vivo* siRNA retention following intradiscal injection of si*Rpt*@cGNS or si*Rpt*@Invivo. **E** Quantification of background-subtracted fluorescence intensity shown in **C**, normalized to the Day 0 value for each animal (*n* = 3 biologically independent animals, mean ± s.d.). **F** Quantification of background-subtracted fluorescence intensity shown in **D**, normalized to the Day 0 value for each animal. Individual animal trajectories are shown as dotted lines, and group means ± s.d. are shown as solid lines with error bars (*n* = 3 biologically independent animals per group). *p < 0.05, **p < 0.01. **G** Fluorescence images showing cellular localization and intradiscal retention of siRNA following treatment with si*Rpt*@cGNS or si*Rpt*@Invivo. Scale bars, 100 μm; enlarged images, 50 μm. **H** Quantification of siRNA-positive cells in the NP, NP–AF border, and AF regions shown in **G** (*n* = 4 biologically independent animals per group at each time point, mean ± s.d.). Statistical significance in **E** was determined using one-way repeated-measures ANOVA followed by Dunnett's multiple comparison test. Data in **F** were analyzed using two-way repeated-measures ANOVA followed by Sidak's multiple comparison test. Data in **H** were analyzed using two-way ANOVA followed by Sidak's multiple comparison test.

We then longitudinally monitored Cy5-labeled cGNS after intradiscal injection using the *in vivo* imaging system (IVIS) (Fig. 7B). cGNS-associated fluorescence signal declined rapidly within the first 24 h, followed by a slower decrease over time. The signal remained detectable for up to 84 days post-injection, indicating a prolonged persistence within the disc (Fig. 7C–E). No apparent body weight loss was observed following intradiscal cGNS administration, and hematoxylin and eosin (H&E) staining of major organs showed no obvious pathological abnormalities after administration of either empty cGNS or si*Rpt*@cGNS (Fig. S11A and B).

To compare *in vivo* retention, Cy5-labeled siRNA was delivered as either si*Rpt*@cGNS or Invivofectamine® 3.0-based *Rptor* siRNA complexes (si*Rpt*@Invivo). IVIS imaging demonstrated a markedly more prolonged intradiscal Cy5 signal in the si*Rpt*@cGNS group than in the si*Rpt*@Invivo group (Fig. 7D–F). Fluorescence decay analysis further showed a longer apparent fluorescence half-life in the si*Rpt*@cGNS group than in the si*Rpt*@Invivo group, defined as the time required for the normalized signal to decline to 50% of the Day 0 value (22.9 versus 4.6 days).

Fluorescence imaging of cryosections further revealed a sustained intracellular Cy5-associated signal in the disc NP region of the si*Rpt*@cGNS group for up to 12 weeks, with Cy5-positive granules predominantly localized within the cytoplasm of NP cells. In contrast, si*Rpt*@Invivo initially showed a more diffuse distribution across the disc NP and AF, and intracellular Cy5 fluorescence in the NP was markedly reduced by Day 56 (Fig. 7G and H). Regional quantification further demonstrated that the percentage of Cy5-positive cells was significantly higher in the si*Rpt*@cGNS group than in the si*Rpt*@Invivo group within the NP region at all evaluated time points. In the NP–AF border region, a significant difference was observed at 4 weeks, whereas no significant between-group difference was observed in the AF region (Fig. 7H). These findings indicate that cGNS promote a prolonged and preferential cellular retention of siRNA-associated fluorescence within the disc NP and adjacent NP–AF border.

### 2.7 *In vivo* si*Rpt*@cGNS supports disc structure preservation with the suppression of apoptosis, senescence, and ECM catabolism

To evaluate the *in vivo* therapeutic efficacy of si*Rpt*@cGNS in a mechanically induced degeneration model, we used a previously established rat tail temporary static compression system [49,50] (Fig. 8A and Fig. S12A). si*Rpt*@cGNS was injected into Co8–Co9 and Co11–Co12 discs, siCtrl@cGNS into Co9–Co10 and Co12–Co13 discs, and PBS into Co13–Co14 discs. Seven days later, static axial compression at 1.3 MPa was applied to Co8–Co9 and Co9–Co10 discs for 24 h and subsequently released. This within-animal design minimizes individual variability and age-related effects [49,50]. In an independent injection-free validation cohort, normalized DHI decreased significantly from 4 to 8 weeks and from 4 to 12 weeks after load release (both *p* < 0.001), with comparable changes in Co8–Co9 proximal and Co9–Co10 distal loaded discs, indicating a progressive radiographic loss of disc height after load release (Fig. S12B and C). Although baseline DHI tended to decrease caudally, it did not differ significantly between the two loaded comparison levels (Co8–Co9 and Co9–Co10) or among the three unloaded control levels (Co11–Co12, Co12–Co13, and Co13–Co14) (Fig. S12D).

**Fig. 8:**
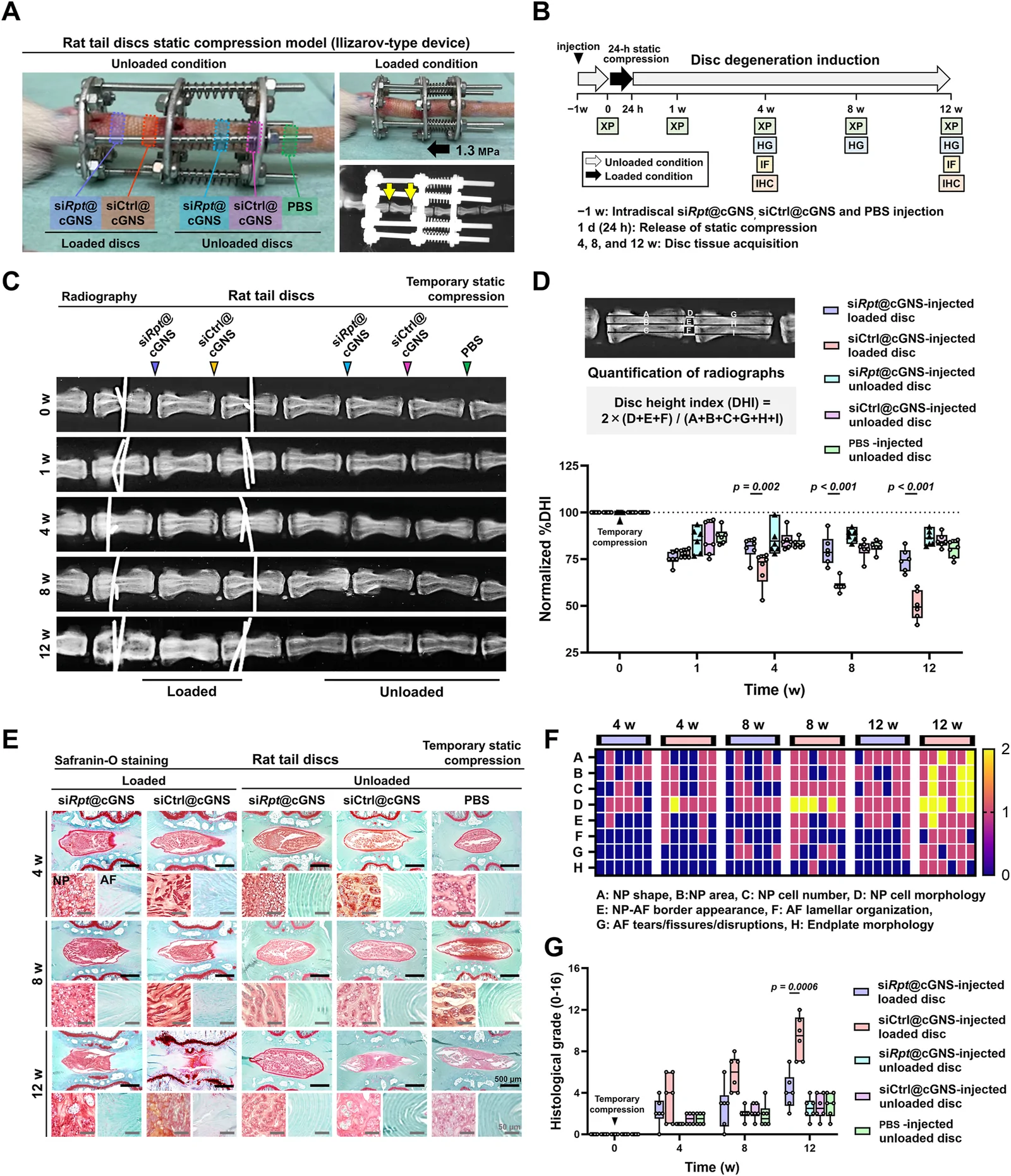
**Preventive effects of si*Rpt*@cGNS against mechanical stress-induced intervertebral disc degeneration in a rat tail model**. **A** Schematic illustration of a rat tail intervertebral disc degeneration model using an Ilizarov-type static compression system. si*Rpt*@cGNS was injected into Co8–Co9 and Co11–Co12 discs, siCtrl@cGNS into Co9–Co10 and Co12–Co13 discs, and PBS into Co13–Co14 discs. Co10–Co11 served as the non-injected control. Static axial compression (1.3 MPa) was applied for 24 h under loaded conditions, generating symmetric craniocaudal compressive stress (yellow arrows). **B** Experimental timeline for intradiscal injection, mechanical loading, and evaluation. **C** Radiographic images showing temporal changes in intervertebral disc morphology following compression. **D** Quantification of DHI shown in **C** (*n* = 6 biologically independent animals at each time point, mean ± s.d.). **E** Safranin O–fast green staining of intervertebral discs at the indicated time points. Scale bars, 500 μm; enlarged images, 50 μm. **F** Heatmap visualization of individual histological degeneration category scores comparing loaded discs across treatment groups. Higher scores indicate more advanced degeneration (*n* = 6 biologically independent animals at each time point). **G** Quantitative histological grading of discs shown in **E** (*n* = 6 biologically independent animals at each time point, mean ± s.d.). Statistical significance in **D** and **G** was determined using two-way mixed-design ANOVA followed by Tukey's multiple comparison test.

No differences in radiographic tissue height were observed among the treatment groups in Co11–Co12, Co12–Co13 and Co13–Co14 unloaded discs at any time point (Fig. 8C and D). In contrast, si*Rpt*@cGNS-treated Co8–Co9 loaded discs exhibited a better preservation of disc height from 4 weeks onward than siCtrl@cGNS-treated Co9–Co10 loaded discs (Fig. 8C and D).

Histomorphological assessment by Safranin O–fast green and hematoxylin staining demonstrated a similar pattern [51] (Fig. 8E–G). Although unloaded discs showed no intergroup differences, Co8–Co9 loaded discs treated with si*Rpt*@cGNS showed a significantly improved degeneration score at 12 weeks (Fig. 8E–G). Differences in NP morphology, area, and cell number between si*Rpt*@cGNS-treated and siCtrl@cGNS-treated loaded discs became more evident over time, and changes in NP cellular architecture were apparent at 4 weeks (Fig. 8E and F).

Multiplex immunofluorescence (IF) confirmed effective *in vivo* RPTOR suppression, as shown by significantly fewer RPTOR-positive NP cells in si*Rpt*@cGNS-treated discs at both 4 and 12 weeks (Fig. 9A and B). In loaded discs, si*Rpt*@cGNS significantly reduced the percentage of apoptotic TUNEL-positive cells and senescent p16^INK4A^-positive cells at both 4 and 12 weeks (Fig. 9A and B). Additional IF showed increased LC3 and decreased p62/SQSTM1 accumulation at 4 weeks in si*Rpt*@cGNS-treated loaded discs, confirming sustained autophagy induction (Fig. S13A and B).

**Fig. 9:**
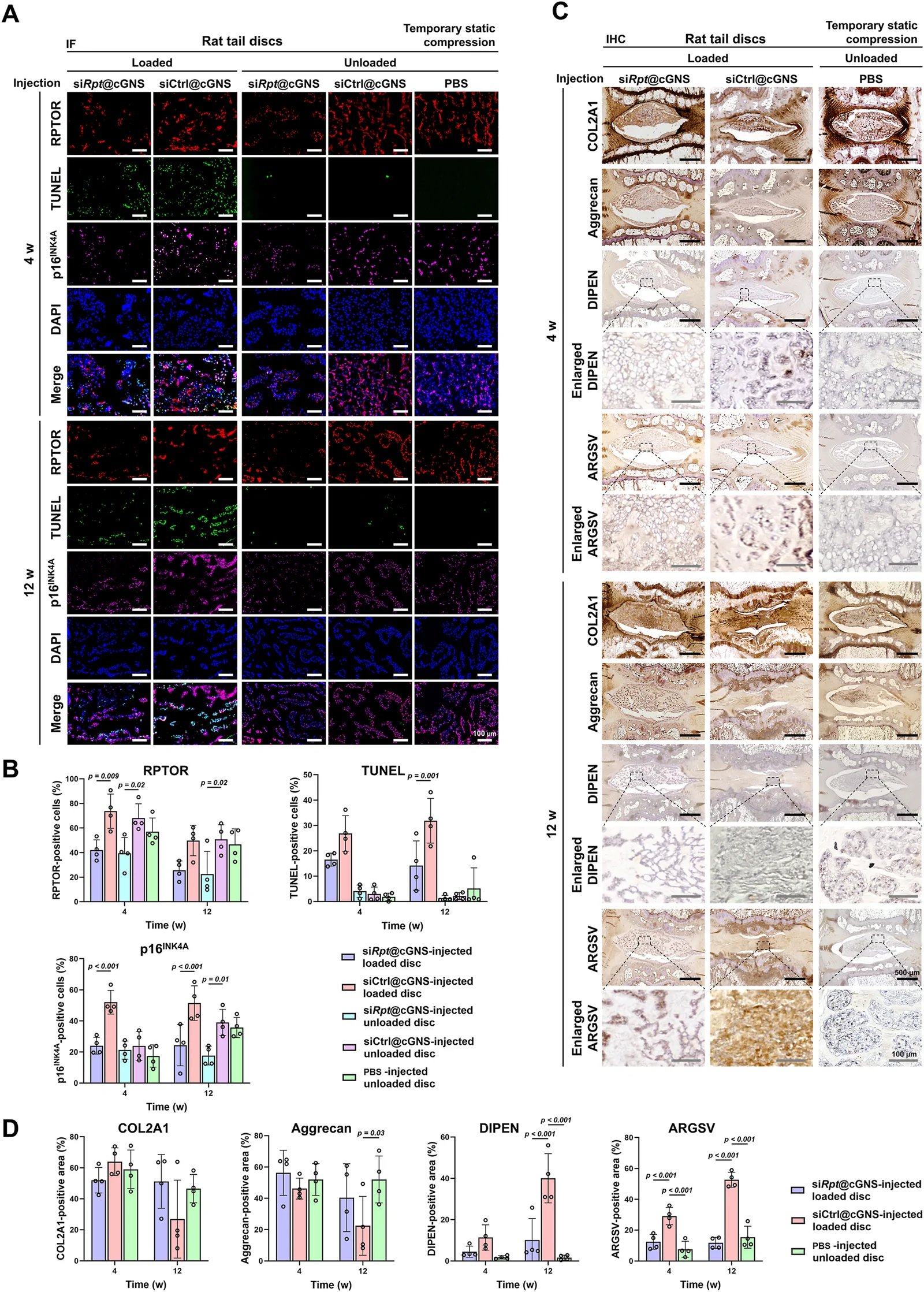
***In vivo* effects of si*****Rpt*****@cGNS on RPTOR expression, apoptosis, senescence, and ECM integrity in mechanically loaded rat intervertebral discs**. **A** IF staining of the NP region showing RPTOR expression, TUNEL-positive apoptotic cells, and p16^INK4A^-positive senescent cells in mechanically loaded and unloaded discs. Scale bars, 100 μm. **B** Quantification of RPTOR-positive, TUNEL-positive, and p16^INK4A^-positive cells shown in **A** (*n* = 4 biologically independent animals, mean ± s.d.). **C** IHC staining of whole intervertebral discs for aggrecan, COL2A1, and the aggrecan neoepitopes DIPEN and ARGSV, with enlarged images of the NP region. Scale bars, 500 μm; enlarged images, 100 μm. **D** Quantification of immunopositive areas shown in **C** (*n* = 4 biologically independent animals, mean ± s.d.). Statistical significance was determined using one-way ANOVA followed by Tukey's multiple comparison test.

Moreover, immunohistochemistry (IHC) for ECM integrity supported these findings (Fig. 9C and D). At 4 weeks, loaded discs treated with si*Rpt*@cGNS demonstrated reduced aggrecan fragments of catabolic MMP-cleaved DIPEN and ADAMTS-cleaved ARGSV (Fig. 9C and D). At 12 weeks, si*Rpt*@cGNS-treated discs further presented the reduction in these aggrecan degradation products together with maintained COL2A1 and aggrecan expression within the NP region (Fig. 9C and D). Consequently, intradiscal delivery of si*Rpt*@cGNS enhances autophagy while suppressing apoptosis, senescence, and ECM catabolism *in vivo*, accompanied by preservation of disc structure in mechanically stressed discs.

Finally, to assess the therapeutic potential beyond the prophylactic setting, we used a delayed treatment model in which disc degeneration was first induced and siRNA was injected 4 weeks later (Fig. S14A). Sixteen weeks after degeneration induction, corresponding to 12 weeks post-injection, radiographic analysis showed a significantly improved disc height preservation in the si*Rpt*@cGNS group compared with the siCtrl@cGNS group (Fig. S14B and C). Histological grading likewise showed a superior disc structural preservation with reduced degeneration in the si*Rpt*@cGNS group (Fig. S14D–F). Therefore, si*Rpt*@cGNS exerts disc-preserving effects in both preventive and post-traumatic settings against degeneration.

We additionally performed an *in vivo* pilot study using intradiscal temsirolimus to examine whether a single local administration of a pharmacological mTORC1 inhibitor could similarly protect against disc degeneration (Fig. S15A). Under the conditions tested, temsirolimus did not significantly preserve disc height or improve histological degeneration compared with the vehicle group (Fig. S15B–F). These findings suggest that a single administration of temsirolimus is insufficient to achieve a significant disc-protective effect in this model, providing the rationale for developing sustained RNAi delivery platforms.

## 3 Discussion

In this study, we established a cGNS-based RNAi platform incorporating siRNA targeting *Rptor*, which encodes an essential component of mTORC1, to achieve sustained gene silencing in the intervertebral disc. Fluorescence analyses demonstrated a prolonged retention of cGNS/siRNA-associated signals, whereas direct measurements of RPTOR confirmed a sustained target suppression. Functionally, si*Rpt*@cGNS enhanced autophagy, suppressed apoptosis and senescence, and inhibited ECM catabolism in disc NP cells *in vitro* and *in vivo*. Disc protection was observed in preventive and delayed-treatment settings, supporting the therapeutic potential of si*Rpt*@cGNS even when administered after the onset of degeneration.

The intervertebral disc is an avascular tissue, in which oxygen and nutrient supply depend primarily on diffusion through cartilaginous endplates [10]. Endplate calcification, microcirculatory disturbances, and mechanical overload can easily impair the physiologically low distribution, exposing disc cells to hypoxic and nutrient-deprived conditions that promote apoptosis and senescence [52]. These cellular changes accelerate ECM degradation and structural collapse. Once degeneration exceeds a critical threshold, the intrinsic reparative capacity of the disc becomes extremely limited, leaving surgical interventions including discectomy and spinal fusion as the principal option for patients with persistent symptoms and advanced disease. Given this hostile and poorly reparative microenvironment, therapeutic strategies for disc degeneration should prioritize preserving the survival and function of residual cells rather than merely increasing the number. Although cell-based therapies, *e.g.*, stem cell transplantation, have shown promise, major challenges remain in achieving long-term engraftment and stable therapeutic phenotype [53]. Therefore, targeting intrinsic stress-response pathways to maintain disc-cell survival and homeostasis under degenerative conditions may represent a more rational and durable therapeutic approach.

mTORC1 is a central regulator of cellular adaptation to nutrient and growth conditions. It integrates nutrient and growth signals through the PI3K–AKT–mTOR pathway to promote protein synthesis and suppress autophagy, with RPTOR serving as its essential scaffolding subunit. Supporting the relevance of this pathway to human disc degeneration, five independent human disc transcriptomic datasets showed a concordant enrichment of the mTORC1-associated transcriptional program in degenerated discs [16]. Previous studies have also demonstrated that pharmacological mTORC1 inhibitors, including rapamycin and temsirolimus, suppress inflammation-induced and nutrient deprivation-induced apoptosis, senescence, and ECM catabolism in disc NP cells and reduce MMP13 and ADAMTS5 expression in cartilage [23,25,26,54,55]. Despite these protective effects, pharmacological mTORC1 inhibitors transiently inhibit the activity of existing proteins, allowing pathway reactivation after drug withdrawal. This limitation is particularly relevant in the intervertebral disc, where repeated administration is undesirable and local drug diffusion and clearance can shorten the duration of exposure. Rapamycin derivatives also have limited water solubility and may cause systemic off-target and immunosuppressive effects [56,57]. By contrast, siRNA suppresses target expression at the mRNA level, enabling the upstream and potentially more sustained regulation of the pathway [58,59]. These considerations provided the rationale for combining *Rptor* silencing with the controlled intracellular delivery properties of cGNS. Mechanistically, mTORC1 inhibition can drive p70S6K-mediated negative feedback and increase AKT phosphorylation [24,25]. In our study, while RPTOR suppression reduced the phosphorylation of p70S6K and also ULK1 at Ser757, thereby enhancing autophagic flux, AMPKα and GSK3β phosphorylation remained unchanged. Together, these findings provide a direct biochemical link between RPTOR suppression, selective mTORC1 inhibition, and autophagy induction, further supporting a predominant mTORC1–ULK1 mechanism rather than the broad activation of stress-responsive signaling pathways.

Because the avascular intervertebral disc is poorly accessible through systemic delivery and repeated punctures may itself accelerate degeneration [60], effective treatments ideally require a single-dose carrier that protects unstable siRNA and sustains its cytoplasmic availability [61]. Unmodified siRNA is highly susceptible to extracellular degradation, making carrier-mediated protection and cytoplasmic delivery essential. Compared with conventional lipid-based carriers [62], cGNS combine biocompatibility with enzyme-responsive biodegradability and, under our optimized conditions, enable higher-dose siRNA delivery with low cytotoxicity and prolonged target suppression. Consistent with this design rationale, Lipofectamine RNAiMAX induced the strongest RPTOR protein suppression on Day 1, whereas si*Rpt*@cGNS showed the maximum suppression on Day 2 and maintained it over time, when compared at the same siRNA concentration. RT-qPCR analysis showed that *Rptor* mRNA expression remained suppressed following si*Rpt*@cGNS treatment through Day 7, supporting a sustained RNAi effect. To investigate the cellular basis of this prolonged activity, we characterized cellular uptake and intracellular trafficking of cGNS and their siRNA cargo. Our inhibitor experiments indicated that cGNS carrying siRNA were internalized predominantly through macropinocytosis and subsequently trafficked to acidic endolysosomal compartments.

LysoTracker imaging further showed that the fluorescence intensities of siRNA and cGNS gradually declined over 24 h but remained detectable, consistent with a partial protection from rapid endolysosomal degradation or clearance. Notably, siRNA–lysosome colocalization decreased earlier than cGNS–lysosome colocalization, suggesting a biphasic intracellular delivery process. In the first phase, a limited fraction of siRNA may dissociate from cGNS and escape into the cytoplasm, potentially facilitated by proton buffering and endosomal membrane destabilization mediated by the spermine-derived cationic groups of cGNS [63]. In the second phase, cGNS retaining residual siRNA may redistribute more slowly from LysoTracker-positive compartments and serve as an intracellular reservoir, from which the enzymatic degradation of the gelatin backbone may gradually release additional siRNA. This model of early productive siRNA escape followed by delayed reservoir-mediated release is consistent with previous live-cell imaging showing an initial burst of cytosolic siRNA release followed by slower release from endosomal reservoirs [64], as well as studies demonstrating a degradation-dependent, sustained siRNA release from gelatin nanospheres [40,44]. Moreover, because only approximately 1%–2% of internalized siRNA is estimated to reach the cytoplasm in a lipid nanoparticle delivery system [65], even limited endosomal escape may be sufficient to support functional gene silencing. This biphasic delivery process could explain the observed, delayed but prolonged RPTOR suppression by si*Rpt*@cGNS. Continued detection of cGNS and siRNA fluorescence after serial passages further supports their intracellular retention and partitioning into daughter cells [40,43,44,66]. Nevertheless, fluorescence imaging does not directly demonstrate that intact cGNS–siRNA complexes cross the endosomal membrane. Therefore, this biphasic process should be regarded as a proposed mechanistic model rather than an established trafficking pathway.

The sustained intracellular behavior observed *in vitro* was also reflected by the prolonged fluorescence retention *in vivo*. IVIS and histological analyses detected cGNS/siRNA-associated fluorescence within disc NP cells for up to 12 weeks, with a greater persistence than that of the conventional lipid-based carrier and preferential localization to the NP and NP–AF border [67]. Importantly, functional RNAi activity was evaluated independently of fluorescence persistence. At the equivalent siRNA dose, si*Rpt*@cGNS maintained RPTOR protein suppression longer than si*Rpt*@Lipo *in vitro*, and the proportion of RPTOR-positive disc NP cells remained significantly reduced at 4 and 12 weeks *in vivo*. Thus, sustained target suppression was supported by direct RPTOR measurements rather than fluorescence persistence alone. Late fluorescent signals may include degradation products or redistributed material, which are therefore not interpreted as evidence of intact, bioactive siRNA. Previous studies have similarly reported effective intradiscal RNAi for up to 24 weeks *in vivo* [68]. The selection of the *in vivo* model was intended to distinguish degeneration induced by mechanical overload from injury caused by delivering therapeutics. Puncture, tail-looping, and axial-compression models are established approaches that represent different initiating pathologies [69]. Because our efficacy study focused on mechanical overload, we used external compression to induce degeneration independently of the intradiscal injection required for treatment, thereby avoiding a large-gauge annular puncture as the model-defining insult. Previous sustained-release siRNA systems for the disc have primarily been evaluated in puncture-induced models [[70], [71], [72]]. In contrast, after a single 33-gauge injection, the cGNS platform achieved sustained RPTOR suppression in the preventive model and further the structural preservation in preventive and delayed-treatment compression models.

This study demonstrates that the sustained modulation of the mTORC1–autophagy axis could suppress intervertebral disc degeneration. Potential applications include preventing adjacent segment disease after spinal fusion, limiting accelerated degeneration after discectomy, and treating early-onset degeneration associated with conditions such as lumbar spondylolysis [[73], [74], [75]]. Several translational challenges remain, including the confirmation of long-term safety, determination of whether the structural preservation confers biomechanical benefits under physiologically relevant loading, and optimization of delivery conditions for human use. Because *Rptor* mRNA was not measured directly in treated discs and no formal *in vivo* dose-dependent analysis was performed, the minimum effective dose and dose-response relationships governing the magnitude and duration of target suppression remain unknown. Although siCtrl@cGNS served as a carrier-based sequence control, empty cGNS and naked siRNA were not independently evaluated *in vivo* because the limited number of compressed disc levels precluded additional within-animal groups. Future studies should include these controls to distinguish carrier-related from sequence-specific effects. The used rat tail temporary compression model represents accelerated, mechanically initiated degeneration rather than chronic human disease. Although 1.3 MPa approximates transient lumbar loading, continuous loading for 24 h is nonphysiological. Moreover, rat caudal discs retain notochordal cells and do not reproduce cyclic loading or the age-associated, multifactorial pathogenesis of human lumbar degeneration. The findings shown should therefore be interpreted within this model, and minor contributions of the matched 33-gauge injections or their interaction with loading cannot be excluded. Finally, pilot experiments in primary human degenerative NP cells demonstrated an efficient uptake of cGNS-delivered *RPTOR* siRNA and suppression of RPTOR protein expression but were limited in scope. Validation in a larger number of human sample sets and larger-sized animal models is required before clinical translation.

The confined, avascular, and chronically hypoxic intradiscal environment may influence cGNS behavior. Limited fluid exchange may promote retention, whereas mechanical loading and degeneration-associated proteolysis may alter particle distribution and accelerate gelatin degradation and siRNA release [76]. Hypoxia and lactic acidosis are unlikely to degrade gelatin directly but may modify release through cellular and enzymatic responses. Thus, the collagenase-containing *in vitro* assay represents accelerated enzymatic degradation rather than the complex intradiscal environment. Future studies should incorporate hypoxia, physiologically relevant loading, and disease-associated enzyme conditions. The *in vitro* and *in vivo* models used distinct initiating stimuli: IL-1β-induced inflammatory/catabolic stress and temporary mechanical compression, respectively. Nevertheless, compression models also induce apoptosis and ECM catabolism [49,77]. Mechanistic analyses *in vitro* showed that RPTOR suppression inhibited mTORC1 signaling and enhanced autophagy by reducing ULK1 Ser757 phosphorylation, thereby attenuating apoptosis, senescence, and ECM catabolism. Although the complete cascade was not examined *in vivo*, sustained RPTOR suppression was accompanied by autophagy-associated changes and protection against disc cellular and matrix degeneration after loading. Therefore, the observed findings would be consistent with the downstream convergence on the RPTOR–mTORC1–autophagy axis despite different upstream stimuli.

Validation across age-related, mechanically induced, and metabolically driven forms of disc degeneration remains necessary. Conversely, mTORC1 inhibition and autophagy restoration may represent a potential therapeutic strategy across multiple musculoskeletal disorders, including osteoarthritis and rheumatoid arthritis [78]. Because the cGNS platform is not restricted to a single target gene, it may also have the potential for adaptation to other disease-related pathways and provide a versatile framework for non-surgical treatment, although this broader applicability requires further investigation.

## 4 Materials and methods

### 4.1 Ethics statement

All animal experiments were performed under the approval and guidance of the Institutional Animal Care and Use Committee (permission number, date; P190709, 31 July 2019; P241102, 5 November 2024). All procedures involving human participants and human-derived specimens were approved by the Institutional Review Board (B210176, 27 September 2021) and conducted in accordance with the Declaration of Helsinki and applicable Japanese laws and regulations. Written informed consent was obtained from each patient before the sample collection.

### 4.2 Human intervertebral disc transcriptomic analysis

Publicly available transcriptomic datasets of human intervertebral disc tissue comparing degenerated discs with less-degenerated or non-degenerated discs were retrieved from the NCBI Gene Expression Omnibus (GEO). Five datasets were included: GSE23130, GSE165722, GSE244889, GSE199866, and GSE56081 [[79], [80], [81], [82], [83]]. These comprised an Affymetrix microarray dataset (GSE23130), an lncRNA/mRNA array reannotated to RefSeq mRNA (GSE56081), a bulk RNA sequencing dataset (GSE244889), and two single-cell RNA sequencing datasets analyzed using a pseudobulk approach (GSE165722 and GSE199866).

Each dataset was analyzed independently. Differential expression was assessed using the limma package (https://bioconductor.org/packages/release/bioc/html/limma.html, accessed on 1 January 2026) for the microarray, array-based, and bulk RNA sequencing datasets and DESeq2 package (https://bioconductor.org/packages/release/bioc/html/DESeq2.html, accessed on 1 January 2026) for the pseudobulk single-cell datasets. For GSE56081, probe sequences were reannotated to RefSeq mRNA, and gene-level statistics were derived from the resulting limma moderated-*t* table. When multiple probes or features mapped to the same gene symbol, the feature with the smallest *p* value was selected.

GSEA was performed using the fgsea package (https://bioconductor.org/packages/release/bioc/html/fgsea.html, accessed on 1 January 2026). Genes were ranked by their signed effect size, calculated as the log_2_ fold change divided by the standard error, and enrichment was evaluated against HALLMARK_MTORC1_SIGNALING from the MSigDB Hallmark collection retrieved using the msigdbr package (https://bioconductor.org/packages/release/data/experiment/html/msigdb.html, accessed on 1 January 2026). Enrichment was reported as the NES with Benjamini–Hochberg-adjusted *p* values.

A curated panel of representative mTORC1-associated genes (*RPS6, RPS6KB1, EIF4EBP1, EIF4E, EIF4B, HIF1A, SLC7A5, ODC1, VEGFA,* and *SREBF1*) and the upstream activator *RHEB* was used to visualize pathway behavior at the single-gene level. *RPTOR*, encoding the mTORC1 subunit RPTOR, was included as a reference. Genes not represented on a given platform were marked as not detected. All analyses were performed in R using limma, DESeq2, fgsea, and msigdbr, and figures were generated using Matplotlib (https://pypi.org/project/matplotlib/, accessed on 1 January 2026) in Python.

### 4.3 Preparation of cGNS

Gelatin (isoelectric point, 9.0) derived from acid-processed porcine skin (Nitta Gelatin, Japan) was cationized by coupling spermine to its carboxyl groups via a carbodiimide-mediated reaction, as previously described [40,43,44]. This reaction was performed at 40 °C, followed by dialysis against distilled deionized water (DDW) with 10 water changes over 3 days and subsequent lyophilization. The degree of amine substitution was quantified using the TNBS assay [84]. Cationized gelatin was dissolved in DDW and incubated at 40 °C. Nanospheres were formed by acetone-induced coacervation, followed by chemical crosslinking with GA [43]. Briefly, 1.25 mL of aqueous cationized gelatin at 50 mg/mL was preheated to 40 °C. Then, 5 mL of acetone was added to induce coacervation. To crosslink the resulting coacervates, GA at 5–100 μL was added, and the reaction mixture was maintained at 40 °C for 6 h. Unreacted aldehyde groups were quenched by adding 3 mL of 100 mM glycine, followed by overnight agitation at room temperature to allow evaporation of residual acetone. Subsequently, the formed cGNS were recovered by centrifugation at 10,000 × *g* for 15 min at 25 °C and resuspended in DDW. This washing step was repeated three times. Finally, the concentration of suspended cGNS was determined from the weight of freeze-dried cGNS and the corresponding suspension volume.

To assess time-course degradation *in vitro*, cGNS were suspended in PBS with or without collagenase D (11088858001, Roche, Switzerland) and incubated at 37 °C. Turbidity was monitored using UV-visible spectrophotometry (Evolution One Plus, Thermo Fisher Scientific, MA).

### 4.4 siRNA incorporation into cGNS

si*Rpt*@cGNS was prepared by incubating 50 μL of cGNS at 1 mg/mL with 50 μL of *Rptor* siRNA at 20 μM in 150 μL of nuclease-free water under gentle mixing, followed by centrifugation at 11,000*× g* for 15 min at 25 °C to remove unbound siRNA. Loading efficiency was calculated by measuring the amount of RNA remaining in the supernatant using the Qubit fluorometer (Thermo Fisher Scientific). Prepared si*Rpt*@cGNS was resuspended in DDW for subsequent experiments.

For physicochemical characterization of cGNS with or without siRNA incorporation, hydrodynamic diameter and PdI were measured by dynamic light scattering after resuspension in 10 mM PBS at pH 7.4, and the zeta potential was determined by electrophoretic light scattering after resuspension in 10 mM phosphate buffer at pH 7.4 using the Zetasizer Nano ZS analyzer (Malvern Instruments, UK). FITC-labeled and Cy5-labeled cGNS were prepared for imaging according to the manufacturer's protocol (Lumiprobe, MD).

To examine siRNA release *in vitro*, si*Rpt*@cGNS were incubated under the same conditions. siRNA released into the supernatant was quantified using the Varioskan LUX fluorescence spectrophotometer (Thermo Fisher Scientific). The cumulative siRNA release profile was fitted to a one-phase exponential association model with an initial burst term:

$$F\left(t\right)=B+\left(F_{\infty }-B\right)\left[1-e^{-k\left(t-0.5\right)}\right]$$

where F(t) is the cumulative percentage release at time t, B is the initial release at 0.5 h, $F_{\infty }$ is the estimated plateau, and k is the apparent first-order release-rate constant.

### 4.5 Cryo-TEM

Immediately before vitrification, cryo-EM grids were hydrophilized using a JEC-3000FC under standard conditions of 7 Pa and 10 mA for 10 s. Approximately 3 μL of each sample was applied to the carbon side of the grid using a Vitrobot Mark IV under controlled temperature and humidity. After blotting under preset conditions, the grids were immediately plunge-frozen in liquid ethane, transferred to liquid nitrogen, and stored until imaging. Cryo-TEM imaging was performed using a Thermo Scientific Krios G4-OUS01 cryo-transmission electron microscope equipped with a Selectris X Imaging Filter (Thermo Fisher Scientific) at an accelerating voltage of 300 kV.

### 4.6 Gel retardation and heparin displacement assays

To evaluate siRNA complexation with cGNS, a gel retardation assay was performed using samples prepared at the indicated siRNA/cGNS ratios and mixed with RNA loading buffer (182-02571, Fujifilm Wako, Japan) with heat denaturation. Free *Rptor* siRNA was used as the control. For all conditions, the amount of siRNA loaded into each well was adjusted to 40 pmol. Samples were electrophoresed using a denaturing polyacrylamide gel (194-15881, Fujifilm Wako) in Tris-borate-EDTA (TBE) buffer. After electrophoresis, the gels were stained with ethidium bromide, and siRNA bands were visualized using a gel imaging system.

For the heparin displacement assay, si*Rpt*@cGNS was prepared at the standard loading ratio of 20 pmol siRNA per 1 μg cGNS and incubated with increasing amounts of heparin sodium at the indicated heparin/siRNA weight ratios for 30 min at 25 °C. The amount of siRNA loaded into each well was fixed at 40 pmol, corresponding to 2 μg cGNS per well. Samples were then mixed with RNA loading buffer, electrophoresed under the same conditions in TBE buffer, stained with ethidium bromide, and imaged to detect displaced siRNA.

### 4.7 RNAi

*In vitro*, following replacement of DMEM containing 10% FBS with Opti-Minimal Essential Medium (MEM) I (31985070, Thermo Fisher Scientific), disc NP cells were reverse-transfected for 24 h with si*Rpt*@cGNS or negative control siRNA incorporated into cGNS (siCtrl@cGNS) [85]. As a comparison, lipid-based transfection with *Rptor* siRNA (si*Rpt*@Lipo) was also prepared using Lipofectamine™ RNAiMAX (13778030, Thermo Fisher Scientific) according to the manufacturer's instructions. Three independent *Rptor* siRNA sequences were screened, and sequence 2 was selected for subsequent experiments based on its knockdown efficiency (Table S3).

For *in vitro* apoptosis and senescence assays, transfected cells were treated for 96 h with FBS-free DMEM containing recombinant IL-1β at 10 ng/mL (097-07631, Fujifilm Wako).

For the *in vivo* retention experiments, Cy5-labeled *Rptor* siRNA was formulated with Invivofectamine™ 3.0 Reagent (si*Rpt*@Invivo; IVF3005, Thermo Fisher Scientific) according to the manufacturer's instructions and used as a conventional-carrier comparator.

### 4.8 Cells

For *in vitro* experiments, 12-week-old male Sprague–Dawley rats were obtained from SLC, Japan (*n* = 36). Intervertebral discs were harvested from the entire spine following euthanasia. Disc NP tissues were isolated, minced, and individually processed for enzymatic digestion in Dulbecco's Modified Eagle's Medium (DMEM; D5796, Sigma-Aldrich, MO) containing 0.114% type II collagenase (LS004176, Worthington Biochemical, NJ) at 37 °C for 40 min with gentle agitation. Residual suspension was filtered twice through 70-μm cell strainers (Corning, NY) and centrifuged at 1000*× g* for 5 min to collect primary disc NP cells. Cells were cultured in DMEM supplemented with 10% fetal bovine serum (FBS; F2442, Sigma-Aldrich) and 1% penicillin–streptomycin (26253-84, Nacalai Tesque, Japan) at 37 °C in a humidified atmosphere containing 5% CO_2_ and 2% O_2_, to simulate the hypoxic disc environment [11]. Medium was refreshed after 24 h and changed every 2–3 days thereafter. Cells were passaged at approximately 80% confluence, and only first-passage and second-passage rat disc NP cells were used for subsequent experiments [[24], [25], [26],^67^,^86^] (Fig. S2).

For *in vitro* pilot validation experiments, human degenerative NP tissues were obtained from discarded surgical specimens of patients who underwent lumbar discectomy or interbody fusion surgery, with the institutional ethics approval and informed consent. Disc degeneration was assessed using the Pfirrmann grading system on preoperative MRI [87]. Disc NP tissues were minced and digested in DMEM containing 10% FBS, 1% penicillin–streptomycin, and 0.114% type II collagenase at 37 °C for 1 h. After filtration through 70-μm cell strainers and centrifugation at 1000 × *g* for 5 min, isolated cells were cultured in DMEM supplemented with 10% FBS and 1% penicillin–streptomycin at 37 °C under 5% CO_2_ and 2% O_2_. Only first-passage human disc NP cells were used.

### 4.9 cGNS and siRNA uptake assay

*In vitro*, to evaluate cellular uptake, FITC-labeled cGNS and Cy5-labeled *Rptor* siRNA were used. Disc NP cells were seeded in 4-well chamber slides, transfected with si*Rpt*@cGNS or si*Rpt*@Lipo for 24 h, fixed with 4% paraformaldehyde for 10 min, and stained with DAPI-containing mounting medium (H-1800, Vector Laboratories, CA). Intracellular uptake of cGNS and siRNA was visualized using a confocal laser scanning microscope (FV1000, Olympus, Japan). The percentage of cGNS-positive and *Rptor* siRNA-positive cells was calculated relative to DAPI-positive nuclei in duplicated five randomly selected regions of interest (ROIs) using the ImageJ software (https://imagej.net/ij/, accessed on 1 January 2026). For the quantitative analysis of siRNA fluorescence retention, Cy5 fluorescence intensity was measured from identical ROIs under the same imaging conditions, background-corrected, normalized to the number of DAPI-positive nuclei, and further normalized to the Day 1 value.

### 4.10 Cell viability assay

*In vitro*, disc NP cells were seeded in 96-well plates and treated with naked *Rptor* siRNA, unloaded cGNS, siCtrl@cGNS, or si*Rpt*@cGNS; untreated cells served as the control. For comparison of delivery-system cytotoxicity, cells were also transfected with varying concentrations of si*Rpt*@cGNS or si*Rpt*@Lipo. Cell viability was assessed at the indicated time points using a WST-8 assay kit (07553-15, Nacalai Tesque, Japan) according to the manufacturer's instructions. Absorbance was measured at 450 nm using a Model 680 microplate reader (Bio-Rad, CA).

### 4.11 cGNS uptake inhibition assay

*In vitro*, to investigate cellular uptake pathways of cGNS, disc NP cells were pretreated for 30 min with the following endocytosis inhibitors in Opti-MEM: chlorpromazine at 15 μM, sucrose at 45 mM, methyl-β-cyclodextrin at 1 mM, nocodazole at 20 μM, cytochalasin D at 5 μM, wortmannin at 300 nM, or EIPA (5-(N-ethyl-N-isopropyl)amiloride) at 50 μM. As the control, cells were incubated at 4 °C without inhibitors to suppress energy-dependent endocytosis. After pretreatment, cells were transfected with Cy5-labeled si*Rpt*@cGNS. After 24 h, nuclei were counterstained with Hoechst 33342 (H1399, Thermo Fisher Scientific) for 5 min. Then, cellular uptake was evaluated using the BZ-X710 fluorescence microscope (Keyence, Japan). siRNA-positive cells were identified based on the presence of Cy5 fluorescence. The percentage of positive cells was calculated by counting cells in duplicated five random ROIs using ImageJ.

### 4.12 Lysosomal escape assay

*In vitro*, disc NP cells were incubated for 3, 12, or 24 h with either Cy5-labeled cGNS alone or cGNS incorporating Cy5-labeled *Rptor* siRNA. Cells were then stained with LysoTracker Green (L7526, Thermo Fisher Scientific) and Hoechst 33342. Fluorescence images were acquired using a BZ-X710 fluorescence microscope under identical settings across time points within each experiment. Colocalization with LysoTracker-positive compartments was quantified using the Coloc 2 plugin in ImageJ, with Cy5 designated as channel 1 and LysoTracker Green as channel 2. Thresholded Manders’ coefficient tM1 represented the fraction of cGNS or siRNA fluorescence overlapping with LysoTracker-positive compartments. Intracellular Cy5 fluorescence intensity was background-subtracted and normalized to the corresponding 3-h value.

### 4.13 WB

*In vitro*, disc NP cells were lysed on ice in a radioimmunoprecipitation assay buffer (16488-34, Nacalai) supplemented with protease inhibitors (25955-11, Nacalai) and phosphatase inhibitors (P5726 and P0044, Sigma-Aldrich). Lysates were centrifuged at 14,000*× g* for 15 min at 4 °C. Protein concentration was determined using a bicinchoninic acid assay kit (23227, Thermo Fisher Scientific). Equal amounts of protein were mixed with a sample buffer (1610747, Bio-Rad), boiled for 5 min, separated on 5%–15% gradient sodium dodecyl sulfate–polyacrylamide gels (21791-84, Nacalai), and transferred to polyvinylidene difluoride membranes (10600021, GE Healthcare, IL). Membranes were blocked and incubated with 1:200–1:1000 primary antibodies overnight at 4 °C, followed by 1:2000 anti-rabbit and anti-mouse secondary antibodies (NA931 and NA934, GE Healthcare) for 1 h at room temperature. The primary antibodies used in this experiment are listed in Table S4. Signals were detected using enhanced chemiluminescence and captured using the iBright CL1500 Imaging system (Thermo Fisher Scientific). Band intensity was quantified using ImageJ. Protein expression was normalized to tubulin. In this study, expression and phosphorylation of disc NP phenotype-related, PI3K–AKT–mTOR signaling-related, autophagy-related, apoptosis-related, and senescence-related components, markers, and substrates were measured.

### 4.14 RT–qPCR

*In vitro*, total RNA was isolated using the RNeasy Mini kit (74104; Qiagen, Germany) according to the manufacturer's protocol. RNA concentration and purity were assessed using a NanoDrop One spectrophotometer (Thermo Fisher Scientific). Complementary DNA (cDNA) was synthesized from total RNA using the High-Capacity cDNA Reverse Transcription kit (4368814, Thermo Fisher Scientific). RT–qPCR was performed using TaqMan Universal PCR Master Mix (4304437, Thermo Fisher Scientific) on the ABI Prism 7500 RT–PCR system (Thermo Fisher Scientific). Gene-specific TaqMan probe/primer sets are listed in Table S5. RT–qPCR was performed in duplicate, and relative target gene expression was normalized to *Gapdh* and calculated using the ΔΔCt method [88].

### 4.15 Autophagy assay

*In vitro*, autophagic flux was monitored by WB for LC3-I/II and p62/SQSTM1 as well as by IF for the number of LC3 puncta per cell. In addition, LC3 turnover assay was performed by using BafA1 (54645S, Cell Signaling Technology, MA), a lysosomal inhibitor [46]. Intracellular LC3 puncta were counted using ImageJ.

### 4.16 TUNEL assay

*In vitro*, apoptotic cell death was assessed using a fluorescein-conjugated TUNEL assay kit (MK500, Takara Bio, Japan) according to the manufacturer's instructions. Cells were fixed with 4% paraformaldehyde. Nuclei were counterstained with DAPI. Fluorescence images were acquired using the BZ-X710 fluorescence microscope. The percentage of TUNEL-positive cells was calculated relative to DAPI-positive nuclei in duplicated five random ROIs using ImageJ.

### 4.17 SA-β-gal and intracellular ROS assays

*In vitro*, cellular senescence was analyzed at pH 6.0 using a SA-β-gal staining kit (9860, Cell Signaling Technology, MA) according to the manufacturer's instructions [89]. Cells were fixed. Images were acquired from duplicated five random ROIs via microscope. The proportion of SA-β-gal-positive cells relative to the total cells was determined using ImageJ.

*In vitro*, intracellular ROS-associated fluorescence was assessed using DCFH-DA (340-09811, Dojindo). Following the indicated treatments, cells were washed with PBS and incubated with 10 μM DCFH-DA at 37 °C for 30 min in the dark. Cells were subsequently washed three times with PBS, and fluorescence images were acquired under identical imaging conditions. H_2_O_2_ at 100 μM for 6 h was used as a positive control. DCF fluorescence intensity was quantified using ImageJ and expressed relative to that of the untreated control.

### 4.18 Live–dead assay

*In vitro*, cell viability was measured using a live–dead staining assay kit (22789, AAT Bioquest, CA), according to the manufacturer's instructions. Live and dead cells were visualized using the BZ-X710 fluorescence microscope. The percentage of dead cells was quantified in five randomly selected ROIs using ImageJ.

### 4.19 EdU proliferation assay

*In vitro*, cell proliferation was monitored using an EdU incorporation assay kit (E-CK-A373; Elabscience, TX) according to the manufacturer's instructions. After 6 h of EdU incubation, cells were fixed, counterstained with DAPI, and visualized using the BZ-X710 fluorescence microscope. The proportion of EdU-positive cells was quantified in five randomly selected ROIs using ImageJ.

### 4.20 Animals

For *in vivo* experiments, 12-week-old male Sprague–Dawley rats were used (*n* = 182, weight 381.3 ± 35.0 g). Animals were housed individually under specific pathogen-free conditions with ad libitum access to food and water under a 12-h light/dark cycle at a controlled temperature of 23 ± 2 °C and humidity of 55% ± 5%. Animal health and behavior were monitored throughout the experimental period. No unexpected animal deaths occurred during the study. Additionally, no rats met the humane endpoint criteria requiring euthanasia, including difficulty in eating or drinking, abnormal posture, self-injurious behavior, or more than 20% body weight loss.

Longitudinal measurements of the body weight of rats with intradiscal cGNS injection or untreated control were performed to assess the potential general health toxicity. In addition, H&E staining of the heart, kidney, liver, lung, and spleen collected from rats with intradiscal injection of cGNS, si*Rpt*@cGNS, or untreated control was conducted at postoperative 4 weeks to observe the potential systemic violation out of the disc.

### 4.21 Contrast medium analysis

*In vivo*, the iodinated contrast medium (240 mg I/mL; 00407-1412-10, GE Healthcare) was injected intradiscally at volumes of 1.0–5.0 μL into rat tail discs from Co7–Co8 to Co13–Co14. Immediately after injection, micro-CT (R_mCT2 FX system, Rigaku, Japan) was performed to evaluate the signal volume within the NP region at a radiographic energy of 50 kVp and tube current of 200 μA, yielding a voxel size of 15 μm using a Rigaku system. Under general anesthesia and fluoroscopic guidance, the contrast agent was delivered to the disc center using a needle through a 5-mm longitudinal skin incision [48,68]. A 33G needle and an injection volume of 2 μL per disc were used to minimize injection-related injury. The needle tip was positioned within the nucleus pulposus without penetrating the contralateral annulus. After injection, the needle was retained in position for 3 min before withdrawal to reduce reflux through the needle tract. Identical needle size, puncture procedure, and injection volume were applied to all treatment and control discs [60,[90], [91], [92]].

### 4.22 IVIS

For *in vivo* cGNS degradation, 2-μL suspension containing 6 μg of Cy5-labeled cGNS was injected into the NP region of the rat tail Co10–Co11 disc. Fluorescence signals were acquired at predetermined time points for up to 12 weeks using Lumina III IVIS (PerkinElmer, IL). For *in vivo* siRNA release, si*Rpt*@cGNS and Invivofectamine® 3.0-based *Rptor* siRNA complexes (si*Rpt*@Invivo; IVF3005, Thermo Fisher) containing Cy5-labeled siRNA were injected into rat tail Co8–Co9 and Co10–Co11 discs at a dose of 120 pmol siRNA per disc. Fluorescence signals were measured using regions of interest placed over the injected discs. Background signals were measured from adjacent non-injected tail regions in the same animal at each time point and subtracted from the disc ROI signals. The background-subtracted fluorescence signal was normalized to the Day 0 value for each animal. The apparent half-life was defined as the time at which the normalized fluorescence signal decreased to 50% of the Day 0 value and was calculated via linear interpolation between adjacent time points.

### 4.23 Animal models of intervertebral disc degeneration

For *in vivo* prophylactic effects of cGNS-mediated RNAi, local injection of si*Rpt*@cGNS into rat tail Co8–Co9 and Co11–Co12 discs, siCtrl@cGNS into Co9–Co10 and Co12–Co13 discs, and PBS into Co13–Co14 disc was performed using a 33G needle via a 5-mm skin incision. One week after delivery, an Ilizarov-type external fixation device was applied between Co8 and Co10 vertebrae (Fig. S12A). Animals were anesthetized only during device placement and initiation of compression, and anesthesia was not maintained during the 24-h compression period. Static axial compression at 1.3 MPa was applied to Co8–Co9 and Co9–Co10 discs for 24 h, after which the load was released. The 24-h duration was selected based on a prior calibration study showing persistent radiographic, histological, cellular, and matrix-metabolic changes after load release [49]. The loading magnitude approximates transient lumbar loading during moderate lifting, whereas continuous loading for 24 h is nonphysiological [49,67,93,94]. An independent injection-free cohort underwent the same 24-h compression and was assessed radiographically at 4, 8, and 12 weeks after release to evaluate the severity of disc height loss (Fig. S12B and C). Degenerative changes in the treatment experiments were longitudinally evaluated for up to 12 weeks using radiographic, histological, and molecular analyses (Fig. 8B).

For *in vivo* therapeutic effects even during established degeneration, mechanical loading was first used, followed by intradiscal injection of si*Rpt*@cGNS or siCtrl@cGNS after 4 weeks. Degenerative changes were longitudinally tracked for up to 12 weeks after injection radiographically and histologically (Fig. S14A).

Additionally, to evaluate *in vivo* preventive effect of pharmacological mTORC1 inhibition, temsirolimus (10 μM, 2 μL in saline containing 0.02% DMSO) was intradiscally injected into Co8–Co9 discs, whereas vehicle was injected into adjacent Co9–Co10 discs [55]. Seven days after injection, both levels were subjected to 1.3-MPa static compression for 24 h, followed by radiographic and histological evaluations up to 56 days after compression (Fig. S15A).

### 4.24 Radiography

Lateral radiographs were acquired using a VPX-30E system (Toshiba Medical Supply, Japan) with IXFR film (Fujifilm) using the following settings: exposure time, 30 s; source-to-image distance, 40 cm; tube current, 3 mA; and tube voltage, 35 kV. Intervertebral disc height and vertebral body length were measured at the dorsal, midline, and ventral regions in the sagittal plane, and the average values were used to calculate DHI as previously described [36] (Fig. 8D).

### 4.25 Frozen tissue and paraffin-embedded tissue preparation

Functional spinal units were harvested after euthanasia and fixed *en bloc* in 4% paraformaldehyde (163-20145, Fujifilm Wako) for 24 h. Specimens were decalcified in 10% ethylenediaminetetraacetic acid (2670-4, Ricca Chemical, TX) for 4–6 weeks, embedded in paraffin, and sectioned in the midsagittal plane at 8 μm using the RM2245 rotary microtome (Leica Biosystems, Germany) for histomorphology, IF, and IHC.

Similarly, functional spinal units were embedded in the optimal cutting temperature compound (4583, Sakura Finetek, Japan), snap-frozen in liquid nitrogen, and sectioned in the midsagittal plane at 10 μm using the CM3050S cryostat (Leica Biosystems) to evaluate the cellular localization and intradiscal retention of siRNA.

### 4.26 Histomorphology

Safranin O–fast green and hematoxylin staining was used for disc tissue morphology. Histological degeneration was graded using the Orthopaedic Research Society spine section scoring system [51] (Table S6). Evaluation was performed independently by three blinded examiners in duplicate at a 1-week interval, and the median score was accepted.

### 4.27 IF and IHC

*In vivo*, multicolor IF was conducted to analyze disc intracellular signaling. After deparaffinization, rehydration, antigen retrieval, and blocking, sections were incubated overnight at 4 °C with primary antibodies against RPTOR and p16^INK4A^ (Table S4), together with fluorescein-labeled TUNEL reagent. Additional sections were also incubated with primary antibodies against LC3 or p62/SQSTM1 (Table S4). Subsequently, 1:200 Alexa Fluor 568-conjugated and 647-conjugated secondary antibodies (A-11003 and A-31573, Thermo Fisher Scientific) were applied for 1 h at room temperature. Nuclei were counterstained with DAPI. Image acquisition and positive cell counting were performed similarly using ImageJ.

*In vivo*, IHC was performed to assess disc matrix degradation. Sections were deparaffinized and rehydrated, followed by antigen retrieval. Endogenous peroxidase activity was quenched with 3% hydrogen peroxide and nonspecific binding was blocked with 10% normal goat serum. Sections were incubated overnight at 4 °C with primary antibodies against aggrecan, COL2A1, MMP-cleaved aggrecan DIPEN fragments, and ADAMTS-cleaved aggrecan ARGSV fragments (Table S4), followed by incubation with secondary antibodies (424131 and 424141, Nichirei Biosciences, Japan) using an avidin–biotin–peroxidase complex method. Signals were visualized with DAB (425011, Nichirei Biosciences) and counterstained with hematoxylin (3002-2, Muto Pure Chemicals, Japan). Positive staining was quantified using ImageJ by threshold-based measurement of the percentage of DAB-positive area in the acquired images.

### 4.28 Statistical analysis

Statistical analyses were performed using GraphPad Prism 10 software (GraphPad Software, MA). The statistical test used for each dataset, multiple comparison correction method, sample size, replicate definition, and error bar definition are provided in the corresponding figure captions. Comparisons between the two groups were performed using an unpaired two-tailed Student's t-test, whereas comparisons among multiple groups or time points were performed using one-way ANOVA, two-way ANOVA, repeated-measures ANOVA, or mixed-effects models, followed by appropriate post hoc multiple comparison tests. Data are presented as mean ± s.d. unless otherwise indicated. A *p* value < 0.05 was considered statistically significant.

## CRediT authorship contribution statement

**Yoshiaki Hiranaka:** Writing – original draft, Visualization, Validation, Software, Resources, Methodology, Investigation, Formal analysis, Data curation. **Takashi Yurube:** Writing – original draft, Visualization, Validation, Supervision, Software, Resources, Project administration, Methodology, Investigation, Funding acquisition, Formal analysis, Data curation, Conceptualization. **Naotoshi Kumagai:** Writing – review & editing, Validation, Investigation, Formal analysis. **Yoshiki Takeoka:** Writing – review & editing, Validation, Investigation, Formal analysis. **Yutaro Kanda:** Writing – review & editing, Validation, Investigation, Formal analysis. **Kohei Kuroshima:** Writing – review & editing, Validation, Investigation, Formal analysis. **Masahiko Furuya:** Writing – review & editing, Validation, Investigation, Formal analysis. **Daisuke Nakagawa:** Writing – review & editing, Validation, Investigation, Formal analysis. **Takahiro Kitano:** Writing – review & editing, Validation, Investigation, Formal analysis. **Akihiro Miyajima:** Writing – review & editing, Validation, Investigation, Formal analysis. **Taichi Washisaka:** Writing – review & editing, Validation, Methodology, Investigation, Formal analysis. **Kenichiro Kakutani:** Writing – review & editing, Validation, Supervision, Funding acquisition. **Ryosuke Kuroda:** Writing – review & editing, Validation, Supervision, Funding acquisition. **Yasuhiko Tabata:** Writing – review & editing, Validation, Supervision, Methodology, Funding acquisition.

## Data availability statement

The datasets generated and/or analyzed during the current study are available from the corresponding author on reasonable request.

## Ethics approval and consent to participate

All animal experiments were performed under the approval and guidance of the Institutional Animal Care and Use Committee at Kobe University Graduate School of Medicine (permission number, date; P190709, 31 July 2019; P241102, 5 November 2024) in accordance with institutional guidelines and relevant regulations for laboratory animals. Animal studies are reported in compliance with the ARRIVE guidelines.

All experiments using human surgical specimens were conducted under the approval and guidance of the Institutional Review Board at Kobe University Graduate School of Medicine (permission number, date; B210176, 27 September 2021) in accordance with the Declaration of Helsinki and applicable laws and regulations of Japan. Written informed consent was obtained from each patient before the sample collection.

## Funding

This work was supported by JSPS KAKENHI, Grant Numbers JP21K09323 (T.Y. and K.Kakutani) and JP24K02565 (T.Y., K.Kakutani, and Y.Tabata) as well as by “Uribo Fund” Kobe University Start-Up Program (T.Y.).

## **Declaration of competing interest**

The authors declare the following financial interests/personal relationships which may be considered as potential competing interests: Takashi Yurube reports a relationship with Surgical Spine, Inc. (Tokyo, Japan) that includes: funding grants. Yoshiki Takeoka reports a relationship with Surgical Spine, Inc. (Tokyo, Japan) that includes: funding grants. Kenichiro Kakutani reports a relationship with Surgical Spine, Inc. (Tokyo, Japan) that includes: funding grants. Takashi Yurube reports a relationship with SMI, Inc. (Nagoya, Japan) that includes: funding grants. Yoshiki Takeoka reports a relationship with SMI, Inc. (Nagoya, Japan) that includes: funding grants. Kenichiro Kakutani reports a relationship with SMI, Inc. (Nagoya, Japan) that includes: funding grants. If there are other authors, they declare that they have no known competing financial interests or personal relationships that could have appeared to influence the work reported in this paper.
