# Supplementary material for: Cationized gelatin nanosphere-mediated release-controlled RNA interference targeting mTORC1 protects against intervertebral disc degeneration
Source: Bioact Mater. 2026 Oct 11;69:335–56. doi: 10.1016/j.bioactmat.2026.09.033 (PMC13678022; doi:10.1016/j.bioactmat.2026.09.033)
Supplement: Supplementary file 1 [file mmc1.docx]

**Cationized gelatin nanosphere-mediated release-controlled RNA interference targeting mTORC1 protects against intervertebral disc degeneration**

**Supplementary information contents**

1. Supplementary Figures: Figs. S1–15

2. Supplementary Tables: Tables S1–6

3. Supplementary Movies: A and B

**Supplementary Figures**

**Fig. S1. GSEA running-enrichment plots for HALLMARK_MTORC1_SIGNALING in the respective human datasets.**

Running enrichment score curves from gene set enrichment analysis (GSEA) against the HALLMARK_MTORC1_SIGNALING Hallmark gene set for each of the five human IVDD datasets (GSE23130, GSE165722, GSE244889, GSE199866, GSE56081). In each panel, genes are ranked by their signed effect size (log2 fold-change / standard error) from high (up-regulated in degeneration) to low; tick marks below each curve indicate the positions of gene set members in the ranked list. The normalized enrichment score (NES) and p value are shown for each dataset. Each curve is colored according to the sign of the NES (red = positive enrichment). All five datasets show positive enrichment, consistent with **Fig. 2A.**

**Fig. S2. Early-passage rat disc NP cells retain the notochordal phenotype.**

Western blotting (WB) for notochordal markers of brachyury and CD24 in rat intervertebral disc nucleus pulposus (NP) cells at passages 1–3 in DMEM with 10% FBS. Both proteins remained detectable across early passages, indicating the preservation of notochordal characteristics in disc NP cells used for subsequent experiments.

**Fig. S3. Screening of *Rptor* siRNA sequences for knockdown efficiency in rat disc NP cells.**

**A** Western blotting (WB) for RPTOR in rat intervertebral disc nucleus pulposus (NP) cells transfected with three independent *Rptor* siRNA sequences in DMEM with 10% FBS. **B** Quantification of RPTOR protein expression normalized to Tubulin (*n* = 4 biologically independent experiments, mean ± s.d.). Statistical significance was determined using one-way ANOVA followed by Dunnett’s multiple comparison test.

**Fig. S4. Characterization of primary human disc NP cells and evaluation of *RPTOR* siRNA uptake and gene silencing.**

**A** Magnetic resonance images (MRI) and bright-field images of primary human intervertebral disc nucleus pulposus (NP) cells isolated from surgical specimens obtained from three patients differing in the age, sex, and Pfirrmann grade. Yellow triangles indicate the intervertebral disc segment from which NP tissues were collected. Scale bars, 100 µm. **B** Fluorescence of primary human disc NP cells treated with cGNS incorporating Cy5-labeled RPTOR siRNA (si*RPT*@cGNS) for 24 h. Intracellular fluorescence signals indicate cellular uptake of the siRNA. Nuclei were counterstained with DAPI. Scale bars, 50 µm. **C** Western blotting (WB) for RPTOR in primary human disc NP cells treated with cationized gelatin nanospheres (cGNS) incorporating either of two human-specific *RPTOR* siRNA sequences (si*RPT*@cGNS). Tubulin was used as a loading control.

**Fig. S5. Time-dependent intracellular localization and fluorescence intensity of cGNS in rat disc NP cells.**

**A** Rat intervertebral disc nucleus pulposus (NP) intracellular localization of cGNS and lysosomes at 3, 12, and 24 h. cGNS are shown in red, lysosomes in green, and nuclei in blue. The boxed regions are shown at higher magnification. Scale bars, 50 µm; enlarged images, 10 µm. **B** Quantification of the fraction of cGNS colocalized with lysosomes using Manders’ tM1. **C** Relative cGNS fluorescence intensity at 3, 12, and 24 h. Values were normalized to the corresponding value at 3 h, which was set to 100% (n = 6 biologically independent experiments, mean ± s.d.). Statistical significance was determined using one-way repeated-measures ANOVA followed by Tukey’s multiple comparison test.

**Fig. S6. Evaluation of autophagic flux following *Rptor* silencing in rat disc NP cells.**

**A** Immunofluorescence (IF) for LC3 in rat intervertebral disc nucleus pulposus (NP) cells transfected with control siRNA or *Rptor* siRNA. *Rptor*-silenced cells were treated with bafilomycin A1 (BafA1) or vehicle control dimethyl sulfoxide (DMSO). Nuclei were counterstained with DAPI. Scale bars, 50 µm in the merged images and 10 µm in the enlarged images. **B** Quantification of the number of LC3-positive puncta per cell (*n* = 12 biologically independent experiments, mean ± s.d.). Statistical significance was determined using one-way ANOVA followed by Tukey’s multiple comparison test.

**Fig. S7. Effects of *Rptor* silencing on stress-response signaling and SASP-related gene expression in rat disc NP cells.**

**A** Western blotting (WB) for senescence-associated secretory phenotype (SASP)-related phosphorylated and total GSK3β and AMPKα, as well as BCL2 and BAX in untreated rat intervertebral disc nucleus pulposus (NP) cells and cells treated with cationized gelatin nanospheres (cGNS) incorporating control siRNA (siCtrl) (siCtrl@cGNS) or *Rptor* siRNA (si*Rpt*) (si*Rpt*@cGNS). Tubulin was used as a loading control. **B** Quantification of protein expression normalized to tubulin (*n* = 4 biologically independent experiments, mean ± s.d.). Statistical significance was determined using one-way ANOVA followed by Tukey’s multiple comparison test. **C** Relative mRNA expression of *Pai1* and *Cxcl1* in untreated cells, IL-1β-stimulated cells, and IL-1β-stimulated cells treated with siCtrl@cGNS or si*Rpt*@cGNS. Expression levels were normalized to *Gapdh* and presented relative to the untreated control group (*n* = 3 biologically independent experiments, each assayed in duplicate, mean ± s.d.). Statistical significance was determined using one-way ANOVA followed by Tukey’s multiple comparison test.

**Fig. S8. Evaluation of viability and ROS production following cGNS treatment in rat disc NP cells.**

**A** Viability of rat intervertebral disc nucleus pulposus (NP) cells treated with naked *Rptor* siRNA (si*Rpt*), empty cationized gelatin nanospheres (cGNS), control siRNA (siCtrl)@cGNS, or si*Rpt*@cGNS was evaluated using the water-soluble tetrazolium-8 (WST-8) assay at the indicated time points. Untreated cells served as the control. Cell viability was normalized to that of the control group immediately after transfection (*n* = 4 biologically independent experiments, mean ± s.d.). Statistical significance was determined using two-way ANOVA followed by Dunnett’s multiple comparison test. **B** Fluorescence for intracellular reactive oxygen species (ROS) detected using the dichlorodihydrofluorescein diacetate (DCFH-DA) assay in untreated control cells and cells treated with hydrogen peroxide (H₂O₂), naked si*Rpt*, empty cGNS, siCtrl@cGNS, or si*Rpt*@cGNS. H₂O₂ was used as a positive control. Scale bars, 100 µm. **C** Quantification of ROS-associated DCF fluorescence intensity normalized to that of the untreated control group (*n* = 4 biologically independent experiments, mean ± s.d.). Statistical significance was determined using one-way ANOVA followed by Dunnett’s multiple comparison test.

**Fig. S9. Effects of cGNS-mediated *Rptor* silencing on the viability and proliferation of rat disc NP cells.**

**A** Live–dead staining of untreated rat intervertebral disc nucleus pulposus (NP) cells and cells treated with cationized gelatin nanospheres (cGNS) incorporating control siRNA (siCtrl) (siCtrl@cGNS) or *Rptor* siRNA (siRpt) (siRpt@cGNS) at the indicated time points. Scale bars, 100 µm. **B** Quantification of the percentage of dead cells among total live and dead cells (*n* = 4 biologically independent experiments, mean ± s.d.). **C** Relative total cell counts determined from the live-dead staining images at the indicated time points (*n* = 4 biologically independent experiments, mean ± s.d.). Statistical significance in **B** and **C** was determined using two-way ANOVA followed by Tukey’s multiple comparison test. **D** 5-Ethynyl-2′-deoxyuridine (EdU) incorporation assay showing proliferating untreated disc NP cells and cells treated with siCtrl@cGNS or siRpt@cGNS. Nuclei were counterstained with DAPI. Scale bars, 100 µm. **E** Quantification of EdU-positive cells (*n* = 4 biologically independent experiments, mean ± s.d.). Statistical significance was determined using one-way ANOVA followed by Tukey’s multiple comparison test.

**Fig. S10. Validation of the injection volume, injectability, and tissue distribution of siRpt@cGNS in rat tail discs.**

**A** Micro-computed tomography (CT) evaluation of the distribution of the contrast medium following the injection of increasing volumes into the nucleus pulposus (NP) region of rat tail intervertebral discs. The signal area was quantified relative to the NP area to determine an injectable volume that minimized extravasation (***n* = 3 biologically independent discs, mean ± s.d.**). **B** Phase-contrast and fluorescence images 7 days after injection showing the distribution of Cy5-labeled si*Rpt* delivered using cGNS at doses of 15, 30, 60, and 120 pmol per disc under non-compressed conditions. Corresponding cGNS doses were 0.75, 1.5, 3, and 6 µg, respectively. Injection volume was 2 µL per disc. Scale bars, 100 µm. **C** Photograph showing the injectability of the suspension of cationized gelatin nanospheres (cGNS) incorporating *Rptor* siRNA (siRpt) (siRpt@cGNS) through a microsyringe equipped with a 33-gauge needle. The formulation passed readily through the needle without visible obstruction. **D** Gross photographs of a rat tail intervertebral disc immediately after intradiscal injection of siRpt@cGNS. Red and blue dashed lines indicate the disc NP and annulus fibrosus regions, respectively.

**Fig. S11. Evaluation of body weight and major organ histology following intradiscal cGNS or siRpt@cGNS administration in rats.**

**A** Longitudinal change in the body weight of untreated control rats and rats receiving intradiscal injection of cationized gelatin nanospheres (cGNS) at the indicated time points (*n* = 4 biologically independent animals per group, mean ± s.d.). Statistical significance was determined using two-way repeated-measures ANOVA followed by Tukey’s multiple comparison test. **B** Hematoxylin and eosin (H&E) staining of the heart, kidney, liver, lung, and spleen collected from untreated control rats and rats receiving intradiscal injection of cGNS or cGNS incorporating *Rptor* siRNA (si*Rpt*@cGNS) at postoperative 4 weeks. Scale bars, 500 µm.

**Fig. S12. Establishment and radiographic validation of a rat tail temporary static compression model of intervertebral disc degeneration.**

**A** Schematic illustration of a rat tail static compression model, showing the placement of an Ilizarov-type external fixation device and application of spring-mediated axial compression to generate controlled mechanical loading. **B** Lateral radiographs of loaded and unloaded rat tail intervertebral discs at the indicated time points. Different animals were evaluated at each time point. **C** Disc height index (DHI) values of the cranial and caudal loaded discs at the indicated time points (*n* = 4 biologically independent animals at each time point, mean ± s.d.). Statistical significance was determined using two-way mixed-design ANOVA followed by Sidak’s multiple comparison test. **D** Baseline DHI values of individual rat tail disc segments used in this study (*n* = 4 biologically independent animals, mean ± s.d.). Statistical significance was determined using one-way repeated-measures ANOVA followed by Tukey’s multiple comparison test.

**Fig. S13. *In vivo* autophagy activation in mechanically loaded discs following si*Rpt*@cGNS treatment.**

**A** Immunofluorescence (IF) for LC3 and p62/SQSTM1 was performed in the nucleus pulposus region of mechanically loaded rat tail intervertebral discs treated with cationized gelatin nanospheres (cGNS) incorporating control siRNA (siCtrl; siCtrl@cGNS) or *Rptor* siRNA (si*Rpt*; si*Rpt*@cGNS) at postoperative 4 weeks. Nuclei were counterstained with DAPI. **B** Quantification of LC3- and p62/SQSTM1-positive areas (*n* = 4 biologically independent animals, mean ± s.d.). Statistical significance was determined using an unpaired two-tailed Student’s t-test. Scale bars, 100 µm.

**Fig. S14. Protective effects of si*Rpt*@cGNS in a delayed treatment model of established disc degeneration.**

**A** Experimental timeline illustrating temporary static compression, intradiscal siRNA injection, and subsequent radiographic and histological assessments in a rat tail delayed treatment model of established intervertebral disc degeneration. **B** Lateral radiographs of rat tail mechanically loaded discs treated with cationized gelatin nanospheres (cGNS) incorporating control siRNA (siCtrl) (siCtrl@cGNS) or *Rptor* siRNA (siRpt) (siRpt@cGNS), together with unloaded control discs, at the indicated time points. **C** Quantification of the normalized disc height index (DHI) of loaded discs across treatment groups (*n* = 4 biologically independent animals per group at each time point, mean ± s.d.). **D** Safranin O–fast green staining of rat tail loaded discs treated with siCtrl@cGNS or siRpt@cGNS and unloaded control discs at the indicated time points. Black scale bars, 500 µm; gray scale bars, 50 µm. The nucleus pulposus (NP) and annulus fibrosus (AF) regions are indicated. **E** Heatmap visualization of individual histological degeneration category scores comparing loaded discs across treatment groups. **F** Quantitative histological degeneration grading of loaded discs across treatment groups (*n* = 4 biologically independent animals per group at each time point, mean ± s.d.). Statistical significance was determined using two-way ANOVA followed by Sidak’s multiple comparison test.

**Fig. S15. Effects of temsirolimus in a rat tail temporary static compression model of intervertebral disc degeneration.**

**A** Experimental timeline illustrating intradiscal drug injection, temporary static compression, and subsequent radiographic and histological assessments in a rat tail temporary static compression model of intervertebral disc degeneration. Intradiscal administration of temsirolimus or vehicle control dimethyl sulfoxide (DMSO) was performed 1 week before static compression. **B** Lateral radiographs of rat tail mechanically loaded discs treated with temsirolimus or DMSO, together with unloaded control discs, at the indicated time points. **C** Quantification of the normalized disc height index (DHI) of the loaded discs across treatment groups (*n* = 4 biologically independent animals per group at each time point, mean ± s.d.). **D** Safranin O–fast green staining of mechanically loaded rat tail discs treated with temsirolimus or DMSO at the indicated time points. Black scale bars, 500 µm; gray scale bars, 50 µm. The nucleus pulposus (NP) and annulus fibrosus (AF) regions are indicated. **E** Heatmap visualization of individual histological degeneration category scores comparing loaded discs across treatment groups. **F** Quantitative histological degeneration grading of loaded discs across treatment groups (*n* = 4 biologically independent animals per group at each time point, mean ± s.d.). Statistical significance was determined using two-way ANOVA followed by Sidak’s multiple comparison test.

**Supplementary Tables**

**Table S1. Size, PdI, and zeta potential of cGNS with and without siRNA incorporation.**

| **Materials** | **Apparent size (nm)** | **PdI** | **Zeta potential (mV)** |
| --- | --- | --- | --- |
| cGNS (GA10, µL/mL) | 324.9 ± 7.2 | 0.120 ± 0.038 | 9.03 ± 0.51 |
| cGNS (GA20, µL/mL) | 245.8 ± 1.4 | 0.015 ± 0.052 | 8.90 ± 0.07 |
| cGNS (GA50, µL/mL) | 233.5 ± 4.4 | 0.077 ± 0.004 | 8.62 ± 0.55 |
| cGNS (GA100, µL/mL) | 318.4 ± 3.2 | 0.204 ± 0.011 | 7.69 ± 0.58 |
| Cy5-cGNS (GA20, µL/mL) | 303.4 ± 2.0 | 0.170 ± 0.006 | 4.32 ± 0.40 |
| FITC-cGNS (GA20, µL/mL) | 286.8 ± 5.9 | 0.167 ± 0.010 | 4.24 ± 0.28 |
| siRpt@cGNS (GA20, µL/mL) | 270.0 ± 4.4 | 0.074 ± 0.008 | 4.87 ± 1.29 |
| Data are presented as mean ± s.d. (n = 3).  cGNS, cationized gelatin nanospheres; PdI, polydispersity index; GA, glutaraldehyde; FITC, fluorescein isothiocyanate; Cy5, Cyanine 5. | | | |

**Table S2. siRNA release profile and release kinetics parameters of cGNS-siRNA in collagenase-containing buffer and PBS.**

| **Time (h)** | **Collagenase release (%)** | **PBS release (%)** |
| --- | --- | --- |
| 0 | 0.0 ± 0.0 | 0.0 ± 0.0 |
| 0.5 | 24.7 ± 4.6 | 3.9 ± 5.1 |
| 1 | 27.1 ± 5.3 | 2.8 ± 0.5 |
| 1.5 | 30.4 ± 3.0 | 6.2 ± 0.7 |
| 2 | 36.0 ± 5.5 | 6.4 ± 1.7 |
| 3 | 43.3 ± 10.6 | 6.2 ± 0.7 |
| 6 | 64.1 ± 16.4 | 8.0 ± 1.6 |
| 12 | 83.4 ± 18.8 | 6.3 ± 3.0 |
| 24 | 98.8 ± 19.9 | 11.6 ± 1.1 |
| Data are presented as mean ± s.d. (n = 3). | | |

| **Parameter** | **Collagenase** | **PBS** |
| --- | --- | --- |
| Initial burst release, 0.5 h | 24.7 ± 4.6% | 3.9 ± 5.1% |
| 24 h endpoint / observed plateau | 98.8 ± 19.9% | 11.6 ± 1.1% |
| Initial burst fraction vs 24 h endpoint | 25.0% | 33.5% |
| Fitted plateau, F∞ | 103.3% | Not reliable |
| Release rate constant, k | 0.123 h⁻¹ | Not reliable |
| Half-release time, t1/2 | 4.11 h | Not reliable |
| Model R² | 0.998 | Not reliable |
| Data are presented as mean ± s.d. (n = 3). | | |

**Table S3. siRNA sequences.**

| **siRNA (Rat)** | | | **Sequence (5' to 3')** |
| --- | --- | --- | --- |
| Rptor | Seq. 1 | Sense | CAUGCAUAGCUGUUGUGAtt |
|  |  | Anti-sense | UCAGCAACAGCUAUGCAUGgt |
|  | Seq. 2 | Sense | CAGACUGGAUGAUCAGAUAtt |
|  |  | Anti-sense | UAUCUGAUCAUCCAGUCUGgt |
|  | Seq. 3 | Sense | GUCACCUUGUAGUCCAAUAtt |
|  |  | Anti-sense | UAUUGGACUACAAGGUGACtc |
| Negative control | Sense | | Not applicable |
|  | Anti-sense | | Not applicable |
| For all sequences, Silencer Select Predesigned siRNAs at standard purity were used (Thermo Fisher Scientific). The sequences of Silencer Select Negative Control No. 1 siRNA (4390843; Thermo Fisher Scientific) are proprietary and were not disclosed by the manufacturer.  Rptor, regulatory-associated protein of mTOR complex 1. | | | |

| **siRNA (Human)** | | | **Sequence (5' to 3')** |
| --- | --- | --- | --- |
| RPTOR | Seq. 1 | Sense | ACACCGGACCAUGACGGCUUUCAUU |
|  |  | Anti-sense | AAUGAAAGCCGUCAUGGUCCGGUGU |
|  | Seq. 2 | Sense | GGGUCACUGCCAUGGAGUAUCUGAA |
|  |  | Anti-sense | UUCAGAUACUCCAUGGCAGUGACCC |
| For all sequences, Stealth Predesigned siRNAs at standard purity were used (Thermo Fisher Scientific). | | | |

**Table S4. Primary antibodies.**

| **Antibody** | **Catalog No.** | **Manufacturer** |
| --- | --- | --- |
| RPTOR (rabbit, monoclonal) | 2280 | Cell Signaling Technology (MA) |
| AKT (rabbit, monoclonal) | 4691 | Cell Signaling Technology |
| p-AKT (Ser473) (rabbit, monoclonal) | 4060 | Cell Signaling Technology |
| p70S6K (rabbit, monoclonal) | 2708 | Cell Signaling Technology |
| p-p70S6K (Thr389) (rabbit, monoclonal) | 9234 | Cell Signaling Technology |
| LC3 (rabbit, monoclonal) | 3868 | Cell Signaling Technology |
| p62/SQSTM1 (mouse, monoclonal) | ab56416 | Abcam (UK) |
| Cleaved PARP (rabbit, monoclonal) | 5625 | Cell Signaling Technology |
| Cleaved caspase-9 (rabbit, polyclonal) | 9501 | Cell Signaling Technology |
| p16^INK4A^ (mouse, monoclonal) | sc-1661 | Santa Cruz Biotechnology |
| p53 (mouse, monoclonal) | 2524 | Cell Signaling Technology |
| Aggrecan (mouse, monoclonal) | ab36861 | Abcam |
| COL2A1 (mouse, monoclonal) | sc-52658 | Santa Cruz Biotechnology |
| ARGSV (mouse, monoclonal) | 1042001 | MD Bioproducts (Switzerland) |
| DIPEN (mouse, monoclonal) | 1042002 | MD Bioproducts |
| ULK1 (rabbit, monoclonal) | 8054T | Cell Signaling Technology |
| p-ULK1 (Ser757) (rabbit, monoclonal) | 14202T | Cell Signaling Technology |
| GSK3β (rabbit, monoclonal) | 12456T | Cell Signaling Technology |
| p-GSK3β (Ser9) (rabbit, monoclonal) | 5558T | Cell Signaling Technology |
| AMPKα (rabbit, monoclonal) | 2522S | Cell Signaling Technology |
| p-AMPKα (Thr172) (rabbit, monoclonal) | 2535S | Cell Signaling Technology |
| BCL2 (rabbit, monoclonal) | 28150T | Cell Signaling Technology |
| BAX (rabbit, monoclonal) | 14796T | Cell Signaling Technology |
| Tubulin (mouse, monoclonal) | T9026 | Sigma-Aldrich (MO) |
| RPTOR, regulatory-associated protein of mTOR complex 1; p-AKT, phosphorylated AKT; p70S6K, p70 S6 kinase; p-p70S6K, phosphorylated p70 S6 kinase; ULK1, unc-51-like autophagy-activating kinase 1; AMPKα, AMP-activated protein kinase alpha; GSK3β, glycogen synthase kinase 3 beta. | | |

**Table S5. TaqMan gene expression assays used for quantitative polymerase chain reaction.**

| **Gene** | **Species** | **Assay ID** |
| --- | --- | --- |
| Rptor | Rat | Rn01464431_m1 |
| Il6 | Rat | [Rn01410330_m1](https://www.thermofisher.com/taqman-gene-expression/product/Rn01410330_m1?CID=&ICID=&subtype=) |
| Mmp3 | Rat | [Rn00591740_m1](https://www.thermofisher.com/taqman-gene-expression/product/Rn00591740_m1?CID=&ICID=&subtype=) |
| Mmp13 | Rat | [Rn01448194_m1](https://www.thermofisher.com/taqman-gene-expression/product/Rn01448194_m1?CID=&ICID=&subtype=) |
| Timp1 | Rat | [Rn01430873_g1](https://www.thermofisher.com/taqman-gene-expression/product/Rn01430873_g1?CID=&ICID=&subtype=) |
| Timp2 | Rat | [Rn00573232_m1](https://www.thermofisher.com/taqman-gene-expression/product/Rn00573232_m1?CID=&ICID=&subtype=) |
| Ccl2 | Rat | Rn00580555_m1 |
| Cxcl1 | Rat | Rn00578225_m1 |
| Pai1 | Rat | Rn01481341_m1 |
| Gapdh | Rat | [Rn01775763_g1](https://www.thermofisher.com/taqman-gene-expression/product/Rn01775763_g1?CID=&ICID=&subtype=) |

**Table S6. Histological grade.**

| **Category** | **Feature** | **Score** | **Description** |
| --- | --- | --- | --- |
| NP morphology | NP shape | **0** | Round/oval shape |
|  |  | **1** | Round/oval with mild distortion |
|  |  | **2** | Irregular shape |
|  | NP area | **0** | NP constitutes more than 40% of the disc area |
|  |  | **1** | NP constitutes 20–40% of the disc area |
|  |  | **2** | NP constitutes less than 20% of the disc area |
| NP cellularity | Cell number | **0** | Normal number of cells |
|  |  | **1** | Slight decrease in cell number (cells occupy 1/3–2/3 of the NP space) |
|  |  | **2** | Moderate-to-severe decrease in cell number (cells occupy <1/3 of the NP space) |
|  | Cell morphology | **0** | >80% of NP cells are large and vacuolated |
|  |  | **1** | 30–80% of NP cells are large and vacuolated |
|  |  | **2** | <30% of NP cells are large and vacuolated |
| NP–AF border | Border appearance | **0** | Normal, clear distinction between NP and AF |
|  |  | **1** | Minimal interruption, loss of distinction |
|  |  | **2** | No distinction between NP and AF |
| AF morphology | Lamellar organization | **0** | Discrete, well-organized collagen lamellae |
|  |  | **1** | 20–60% distorted/disorganized lamellae |
|  |  | **2** | >60% distorted/disorganized lamellae |
|  | Tears/fissures/  disruptions | **0** | No ruptured fibers |
|  |  | **1** | Ruptured fibers in ≤1/3 of the annulus |
|  |  | **2** | Ruptured fibers in >1/3 of annulus |
| Endplate | Disruptions & osteophyte/  ossification | **0** | Continuous endplate; no osteophyte/ossification |
|  |  | **1** | Minimal disruption (<1/3); mild osteophyte/ossification |
|  |  | **2** | Moderate/severe disruption (≥1/3); significant osteophyte/ossification |
| NP, nucleus pulposus; AF, annulus fibrosus. | | | |

**Supplementary Movies**

**Supplementary Movie A. Three-dimensional cryo-transmission electron microscopy reconstruction of cationized gelatin nanospheres (cGNS) and cGNS incorporating *Rptor* siRNA (si*Rpt*) (si*Rpt*@cGNS), demonstrating the spherical morphology and spatial organization of nanoparticles.**

**Supplementary Movie B. Injectability of the aqueous suspension of si*Rpt*@cGNS through a 33-gauge needle without visible obstruction.**
